# Transcriptome-based investigation of cirrus development and identifying microsatellite markers in rattan (*Daemonorops jenkinsiana*)

**DOI:** 10.1038/srep46107

**Published:** 2017-04-06

**Authors:** Hansheng Zhao, Huayu Sun, Lichao Li, Yongfeng Lou, Rongsheng Li, Lianghua Qi, Zhimin Gao

**Affiliations:** 1 State Forestry Administration Key Open Laboratory on the Science and Technology of Bamboo and Rattan, International Center for Bamboo and Rattan, Beijing 100102, China; 2 Research Institute of Tropical Forestry, Chinese Academy of Forestry, Guangzhou, 510000, China

## Abstract

Rattan is an important group of regenerating non-wood climbing palm in tropical forests. The cirrus is an essential climbing organ and provides morphological evidence for evolutionary and taxonomic studies. However, limited data are available on the molecular mechanisms underlying the development of the cirrus. Thus, we performed in-depth transcriptomic sequencing analyses to characterize the cirrus development at different developmental stages of *Daemonorops jenkinsiana*. The result showed 404,875 transcripts were assembled, including 61,569 high-quality unigenes were identified, of which approximately 76.16% were annotated and classified by seven authorized databases. Moreover, a comprehensive analysis of the gene expression profiles identified differentially expressed genes (DEGs) concentrated in developmental pathways, cell wall metabolism, and hook formation between the different stages of the cirri. Among them, 37 DEGs were validated by qRT-PCR. Furthermore, 14,693 transcriptome-based microsatellites were identified. Of the 168 designed SSR primer pairs, 153 were validated and 16 pairs were utilized for the polymorphic analysis of 25 rattan accessions. These findings can be used to interpret the molecular mechanisms of cirrus development, and the developed microsatellites markers provide valuable data for assisting rattan taxonomy and expanding the understanding of genomic study in rattan.

Rattan, or climbing palm, represents one of the most important regenerating non-wood forest products, which is second most abundant in tropical forests, particularly in South Asian tropical forests^1^. These palms constitute an integral component of tropical forest ecosystems as well as rural and tribal regions in many tropical countries^2^. Rattans are known for their strength, durability, elasticity and lightness, and they are mainly used for the construction of a variety of furniture products. The world trade of rattan has been estimated to exceed US$2.4 billion, and the annual global revenue trade from furniture produced by rattan has been estimated to exceed US$20 billion^3^.

*Daemonorops jenkinsiana* (Griffith) Martius [*D. margaritae* (Hance) Beccari] is a Chinese-specific rattan with a whip-like cirrus containing hooks and grapnels for climbing, classified in the tribe Calameae, subfamily Calamoideae, and family Arecaceae^4^. The geographic distribution of this palm is concentrated on Hainan island as well as in Guangdong and Guangxi provinces below 23°30′ north latitude^5^. Because of the features of *D. jenkinsiana*, like its attractive physical and mechanical properties, excellent chemical stability, wide product applicability, and edible nature of the shoots as a vegetable, it is considered as one of the most important rattans and presents considerable economic value and wide applicability^6^.

As a typical accompanying plant, *D. jenkinsiana* develops organs that help it climb efficiently within the forest canopy^7^,^8^,^9^. Thus, the cirrus is an essential climbing organ of rattan that consists of a whip-like distal extension of leaf rachis beyond the most distal leaflets^10^. Climbing habits with the aid of cirri and hooks constitute an interesting research focus related to the evolution of climbing strategies^11^. As one of the significant climbing organs, the hooks and grapnels of the cirrus facilitate climbing and development in a forest environment; however, the numerous of them increase the difficulties of cultivating, managing and harvesting rattan. Therefore, a survey on the molecular mechanisms of cirrus development needs to be performed urgently. However, information on the genomics of rattan is currently limited and associations from comparative analyses are not available, which hampers the performance of biological and evolutionary analyses. Furthermore, with the increasing number of identified rattan species, it has become difficult to perform morphology-based taxonomic study based on the reproductive organs due to dioecism and infrequent flowering events, which has led to synopsis in rattan^12^. Therefore, certain molecular markers, such as simple sequence repeats based on transcriptomic data, are crucial for assisting the taxonomic classification of rattan.

Here, we mainly performed transcriptomic sequencing and transcriptome-based *de novo* assembling on four samples of *D. jenkinsiana* from two tissues (cirrus and hook) at two growth stages (initial stage and developed stage) using the Illumina platform. A large number of expressed genes in the deep sequencing pool from the four samples were identified and their functions were annotated. The quality of assembly and the completeness of unigenes were evaluated by RT-PCR experiments and the software of Benchmarking Universal Single-Copy Orthologs (BUSCO), respectively. DEGs were identified through pair-wise comparisons among the cirri at different developmental stages and the significant DEGs involved in developmental pathways, cell wall metabolism, and hook formation were detected in the analysis of cirrus development. Moreover, the loci of microsatellites were identified based on the transcriptomic data, and the developed transcriptome-based SSR markers with polymorphisms were used for molecular marker assisted taxonomy for rattan. The data provided by these analyses may not only provide a fundamental resource for further experimental studies on the function of genes and regulated networks, but also serve as an efficient tool when performing rattan taxonomic classifications.

## Results

### Summary of the transcriptomic data and evaluation of the assembly and unigenes in *D. jenkinsiana*

Four representative samples were collected and then were transcriptome sequencing. These samples included the distal cirrus without hooks at the initial stage (CI) and developed stage (CD) and cirrus with hooks at the initial stage (HI) and developed stage (HD) and two replications for each sample (see Supplementary Fig. S1 and Table S1). Thus, the total of 344,667,614 clean reads were generated in all samples (see Supplementary Table S2), with an average of ~43.08 million reads (~5.21 Gb) per sample. The maximum data size was observed in the HD_rep2 cDNA library, followed by the CD_rep1 cDNA library, whereas the minimum data size was observed in the CI_rep1 cDNA library.

It was noted that we performed the experiment with two replications, not three replications and more, mainly because the low density in hook sample cannot construct high-quality library and it was difficult to collect the initial stage of cirrus in tropical rainforest. Although the previous studies^13^,^14^,^15^,^16^,^17^,^18^,^19^ showed the analysis results based on two biological replicates were acceptable and relatively reliable at some aspects and extents, we had still conducted two investigations of data quality and then approved that data were useful and practical. Our analyses included the calculation of sequence randomness distribution (see Supplementary Fig. S2) and the compared results of the predicted differentially expressed genes (DEGs) based on two sequenced replications and three simulated replications (see Supplementary File 1 for the detailed and Tables S3 and 4).

The result of transcript assembly showed 404,875 transcripts were identified by Trinity software based on ~41.31 million (~4.99 Gb) high-quality reads (see Supplementary Table S5). These transcripts were assembled into 251,468 trinity genes, which identifies transcripts from the same locus. Their length distribution was ranged from 224 bp to 15,633 bp, and an average length of 667 bp. Among them, 39,568 trinity genes were over 1,000 bp. However, in view of the occurrence of low-quality and problematic transcripts in assembly, we thus have assembled and identified 61,569 trinity genes as the unigenes of the rattan by eliminated low-expression and low-coverage transcripts (see Method for the detailed). Furthermore, the statistical result of the unigenes, all protein sequences and nucleotide ones were provided in Supplementary Tables S6 and S7, respectively.

We performed two evaluations (RT-PCR and BUSCO) to respectively estimate the quality of assembled result and the completeness of unigenes. The results showed 76.5% of completed BUSCO was detected, including 72.8% of completed and single-copy ones and 3.7% of completed and duplicated ones. In the remaining result, 12.2% of fragmented BUSCOs and 11.3% of missing one were identified (see Supplementary Table S8). The relatively high proportion of completed BUSCOs indicated the completed unigenes were rather integrity. In the analysis of BUSCO, we used the ‘embryophyta_idb9’ dataset to quantitatively measure for the assessment of transcriptomic completeness based on evolutionarily-informed expectations of gene content. The ‘embryophyta_idb9’ datasets only contain 1440 protein sequences and orthologous group annotations for major clades. Moreover, 12 unigenes were randomly selected and followed by RT-PCR identification. The results depicted the amplicons in the expected size of 10 of the 12 unigenes, and all of the PCR products were confirmed by Sanger sequencing (see Supplementary Table S9). Together with the two evaluation, the relatively accuracy of assembly and the high completeness of unigenes indicated that the results were reliable for further analyses, such as sequence annotation, variation detection and expression analyses.

### Comprehensive annotation of the unigenes

**Figure 1:**
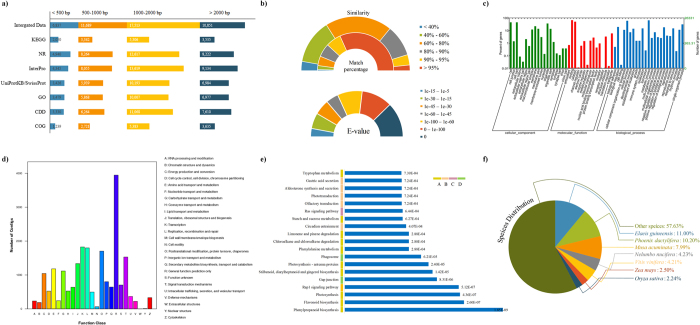
Summary of the sequence annotations. (**a**) BLAST analysis of the non-redundant unigenes against seven public databases. (**b**) Pie-charts showing the distributions of BLAST matches of *D. jenkinsiana* transcriptome unigenes with respect to similarity, match percentage, and e-value. (**c**) GO classification. (**d**) COG annotation of the putative proteins. (**e**) KEGG annotation of the putative proteins. (**f**) Pie-chart showing distributions of the BLAST match of *D. jenkinsiana* transcriptome unigenes with respect to species containing the homologous genes.

Based on the unigenes of the rattan, we performed a series of functional analyses to help researchers to expand the knowledge in rattan by protein sequence similarity searches among seven authorized databases. These databases included the non-redundant NCBI protein database (NR)^20^, UniProtKB/SwissProt database^21^, Clusters of Orthologous Groups (COG) database^22^, Gene Ontology (GO) database^23^, Kyoto Encyclopedia of Genes and Genomes (KEGG) database^24^, Conserved Domain Database for protein classification (CDD)^25^ and the InterPro database^26^. Thus, the integrated result (Fig. 1a) indicated that 46,890 unigenes (76.16%) can translate to relative protein sequences and then these were annotated, whereas the remaining unigenes (23.84%) as non-coding protein sequences were filtered in further analyses. The genes of the missing annotation may be due to the relatively short length of the query sequences and the lack of information on the rattan genome. Moreover, the percentage of the missing annotation (23.84%) by seven authorized databases was higher than the that of BUSCO (11.3%), which was likely caused by different alignment databases.

In Fig. 1b, the results of the BLAST showed the identification of 34,044 unigenes (55.29% of unigenes) returned a BLAST hit above the minimum level of significance and 16,722 unigenes (49.12% of the BLAST hits in Nr database) presenting significant matches (e-values less than 1e^−60^). Moreover, the similarity distributions demonstrated that 68.04% of the sequences presented a similarity greater than 60% and the proportion of matching percentages illustrated that sequences with a greater than 80% match length were abundant. Figure 1c showed the results of the GO classifications for 26,331 sequences, which accounted for 42.77% of unigenes and were categorized into 56 functional groups. The largest number of functional groups and unigenes were found for biological processes, followed by cellular components and molecular functions (see Supplementary Table S10). In Fig. 1d, 16,897 unigenes were annotated and distributed in 24 COG functional categories according to the results of the sequence alignment against the COG database (see Supplementary Table S11). The cluster of ‘general function prediction only’ represented the largest group (3,948 genes, 18.39%), and it was followed by ‘transcription’ (1,826 genes, 8.50%) and ‘replication, recombination and repair functions’ (1,800 genes, 8.38%). In Fig. 1e, we determined that the sequences mapped to the reference authoritative pathways in KEGG database which contained resources for understanding high-level functions and utilities of the biological system. Thus, a total of 14,233 (23.12%) unigenes that presented significant matches in the database were assigned to 287 KEGG pathways. Among the enrichment analysis results (see Supplementary Table S12), the pathway ‘phenylpropanoid biosynthesis’ was the most significant, followed by ‘flavonoid biosynthesis’ and ‘photosynthesis’. Lastly, Fig. 1f showed the results of the species distribution, indicating that the distinct sequences presented top matches with sequences from oil palm (*Elaeis guineensis*) (11.00%), date palm (*Phoenix dactylifera*) (10.20%), banana (*Musa acuminata* subsp. *malaccensis*) (7.99%), and Indian lotus (*Nelumbo nucifera*) (4.23%) (see Supplementary Table S13).

### The analysis of gene expression level and the identification of differentially expressed genes (DEGs)

For the quantification and comparison of the gene expression in different samples, we calculated the fragments per kilobase per million (FPKM) in all samples (see Supplementary Fig. S3a and b). The results showed that the average in each sample was similar and ranged from 4.50 to 5.05. Moreover, the genes with FPKM >= 1 in all of the samples was identified as expressed genes in this study.

**Figure 2:**
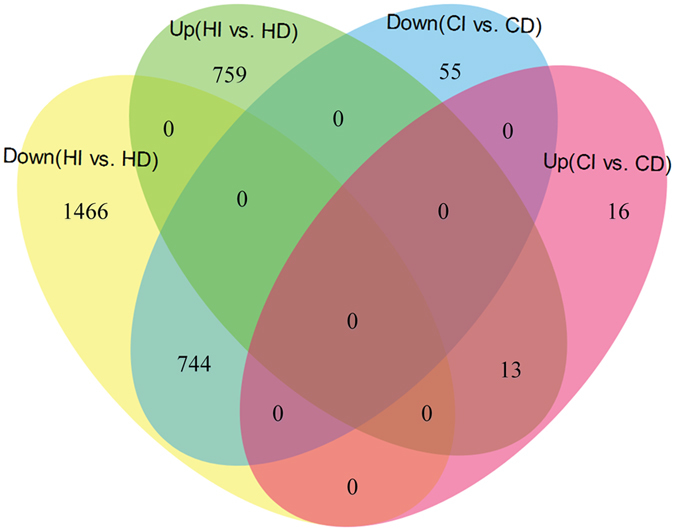
The Venn diagram of the DEG results. Up and Down represents up-regulated genes and down-regulated genes, respectively.

To deeply explore the key genes in cirrus development of rattan, we have identified some DEGs in the cirrus and hook during the developmental stages of *D. jenkinsiana* (see Supplementary Fig. S3c and Table S14–17). The results of the correlation analyses indicated that the replicates were significantly correlated, with the lowest correlation coefficient of 0.8747 observed in the CD sample (see Supplementary Fig. S3d). Moreover, 3,810 genes were identified as significantly enriched or depleted DEGs in the paired comparisons. Twenty-nine genes were up-regulated and 799 genes were down-regulated in the CI *vs*. CD comparison, whereas 772 genes were up-regulated and 2,210 genes were down-regulated in the HI *vs*. HD comparison (Fig. 2).

**Figure 3:**
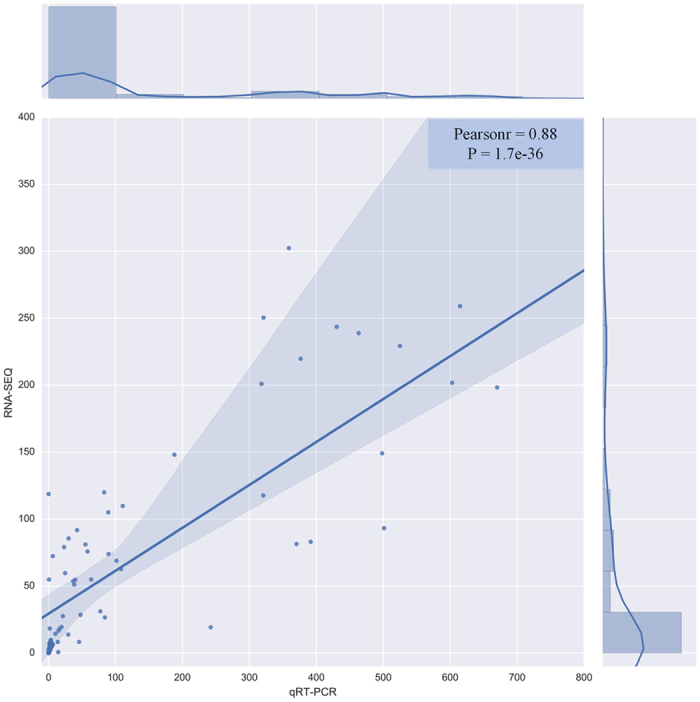
Validation of RNA-Seq data by qRT-PCR. (**a**) Comparison of relative expression of 37 selected genes based on RNA-Seq data and qRT-PCR data. A histogram of gene expression combined RNA-Seq data with qRT-PCR data. X-axes represented 37 selected genes randomly. Y-axes represented relative expression. A: CI sample (Blue box represented qRT-PCR, red box represented RNA-Seq); B: CD sample (Purple box represented qRT-PCR, light blue box represented RNA-Seq); C: HI sample (Dark blue box represented qRT-PCR, dark red box represented RNA-Seq); D: HD sample (Dark purple box represented qRT-PCR, light blue-green box blue box represented RNA-Seq). Error bars indicate standard deviation in both RNA-Seq data and qRT-PCR data. (**b**) Pearson Correlation Coefficient based on expression values between RNA-Seq data and qRT-PCR data.

To experimentally validate the expressed value, quantitative real time PCR (qRT-PCR) assay was performed using the four independently collected tissues, which were in the same developmental stage as those used for the transcriptomic analysis (see Supplementary Fig. S4 and Table S18). As shown in Fig. 3, the Pearson Correlation Coefficient (PCC) based on expression values between qRT-PCR and transcriptomic data among the 37 selected genes randomly was 0.88, indicating that the DEGs observed in transcriptomic data were possibly real because they were confirmed by RT-PCR.

### In-depth investigation of DEGs

To adequately investigate DEGs and facilitate to expand understanding in *D. jenkinsiana*, we had performed enrichment analyses of GO terms and KEGG pathway (see Supplementary Tables S19–S22). We had firstly obtained the individual result of enrichment by selected all DEGs genes as a study dataset and the total genes as background in enrichment analysis. The results indicated that some significant results in both enrichment analyses were detected in ‘structural molecule activity’, ‘non-membrane-bounded organelle’, and ‘intracellular non-membrane-bounded organelle’.

Additionally, we had selected individual DEGs datasets as study datasets to respectively perform enrichment analysis. One interesting dataset was composed of 779 genes from the down-regulated genes in CI *vs*. CD. The result showed the higher significance GO terms were identified in the cellular component category, such as ‘microtubule cytoskeleton’, ‘extracellular region’, and ‘microtubule’ processes, and significantly structure-related terms were involved in the molecular function category. The results of the pathway enrichment indicated the four pathways were relative significant, *i*.*e*. ‘ribosome (ko03010)’ in genetic information processing, ‘gap junction (ko04540)’ and ‘phagosome (ko04145)’ in cellular processes and ‘phenylpropanoid biosynthesis (ko00940)’ in metabolism.

Another interesting dataset was from DEGs in HI *vs*. HD, including 772 up-regulated and 2,210 down-regulated ones. For the up-regulated genes, the GO enrichment results indicated the three significant terms only in the molecular function category, *i*.*e*. ‘glycerophosphodiester phosphodiesterase activity’, ‘catalytic activity’, and ‘transferase activity, transferring glycosyl groups’. For the down-regulated ones, three types of functional genes were focused on cellulose synthase, ribosomal protein and some transcript factors (TFs), and a larger number of proteins involved in cellulose synthase were significantly expressed. The GO enrichment results in the HI *vs*. HD showed that the related metabolic processes of the cell wall were still abundant and highly significant, including ‘secondary cell wall biogenesis’, ‘cell wall organization or biogenesis’, and ‘hemicellulose metabolic process’. Moreover, we found some of ribosomal proteins and certain TFs concentrated on the down-regulated genes of HI *vs*. HD, such as MYB and NAC, which accounted for ~10% of the down-regulated genes.

Lastly, the remaining two DEGs dataset (HD *vs*. CD and CI *vs*. CD) were also analyzed. For the DEGs dataset in HD *vs*. CD, the enrichment result indicated some unique and high significance GO terms were detected in the cellular component category, such as ‘non-membrane-bounded organelle’ and ‘intracellular non-membrane-bounded organelle’ and the GO terms of ‘structural molecule activity’, ‘structural constituent of cytoskeleton’, and ‘cytoskeletal protein binding’ were significant in the molecular function category. In addition, the enrichment results of the DEGs dataset in CI *vs*. CD showed 2,210 down-regulated genes were involved in 187 pathways and were mainly enriched in the three pathways, *i*.*e*. ‘phagosome (ko04145)’ in genetic information processing and both ‘gap junction (ko04540)’ and ‘phagosome (ko04145)’ in cellular processes. We also found some significant pathways in the biosynthesis of secondary metabolites, such as ‘phenylpropanoid biosynthesis (ko00940)’, ‘flavonoid biosynthesis (ko00941)’, and ‘stilbenoid, diarylheptanoid and gingerol biosynthesis (ko00945)’.

### Clustering analysis of DEGs

**Figure 4:**
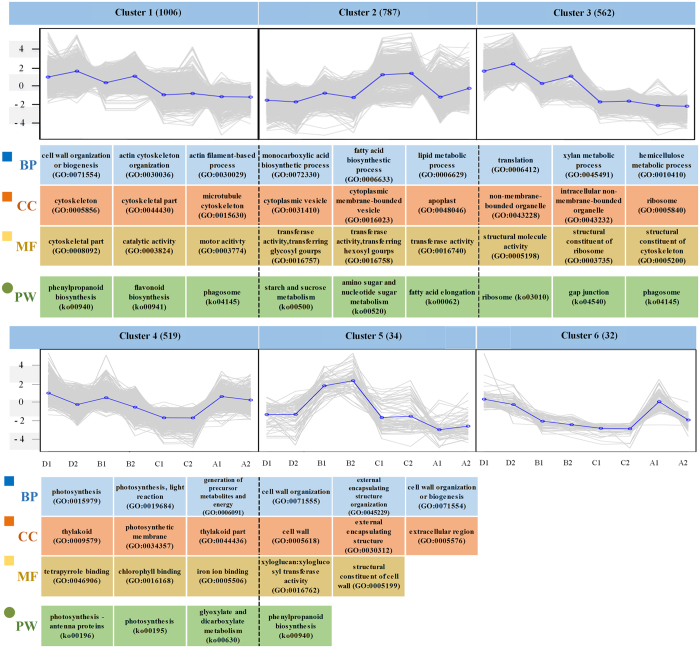
Cluster analysis of the expressed genes in *D. jenkinsiana*. The six groups were identified via the average log_2_(FPKM + 1) value. The number of genes in each group is shown in brackets. The significant GO terms are depicted based on the three GO categories, and the significant KEGG pathways are illustrated. A: CI sample; B: CD sample; C: HI sample; and D: HD sample. 1: replicate 1; 2: replicate 2. BP: biological process, CC: cellular component, MF: molecular function, and PW: KEGG pathway.

We assumed that the same patterns contained similar trends of expressed genes, indicating that these genes may participate in similar or related biological processes. To properly classify the groups of different functional DEGs in further analysis, we performed a clustering analysis based on the DEG expression patterns (see Method and Supplementary Fig. S5). The results indicated all DEGs were clustered into 6 groups, with the gene numbers within the clusters ranging from 32 to 1,006. As shown in Fig. 4, expressed genes of the six groups shared differentially expressed patterns according to the clustering results. The GO term enrichment analyses were also performed using all of the expressed genes as the background to explore the significant GO terms for the expressed genes. The results indicated that certain expressed genes were highly enriched in the cytoskeleton- and actin-related terms for cluster 1 and the transferase- and structure-related terms in the molecular function category of clusters 2 and 3, respectively. In cluster 4, photosynthesis-related terms, such as ‘photosynthesis (GO:0015979)’, ‘thylakoid (GO:0009579)’, and ‘chlorophyll binding (GO:0016168)’, were abundant and reached statistical significance (adjusted *p*-value < 0.05) in the biological processes, cellular components and molecular functions categories. The related GO terms for the cell wall were enriched in cluster 5, including ‘cell wall (GO:0005618)’, and ‘cell wall organization (GO:0071555)’. Furthermore, few or no significant GO terms were identified in clusters 5 and 6 because the data were unable to meet the criteria of GO enrichment.

In the KEGG pathways enrichment analyses of the expressed genes, phenylpropanoid and flavonoid biosynthesis were significant in cluster 1. Carbohydrate and lipid metabolism, including ‘starch and sucrose metabolism’, ‘amino sugar and nucleotide sugar metabolism’, and ‘fatty acid elongation’, were enriched in cluster 2. Ribosomes for genetic information processing as well as gap junctions and phagosomes of cellular processes were detected as higher significance pathways in cluster 3. The result of the pathway enrichment analysis in cluster 4 was similar to that of the GO enrichment analysis and indicated that the pathways for photosynthesis were significant. Similarly, few or no significant GO terms were identified in clusters 5 and 6.

### Identification and development of the biomarkers in rattan (SNP and microsatellite)

Before the analysis of biomarkers, heterozygous rate should be investigated, which facilitated to analyze heterozygous SNPs in further study. Thus, we had obtained genome survey sequence of ~60 Gigabases (Gb) to calculate heterozygous rate and genome size as the genomic background for SNP identification (see Method and Supplementary Table S23). The result showed the heterozygous rate of 1.19 ~ 1.31% and SNP of 0.82% in *D. jenkinsiana* (see Supplementary Table S24 and Fig. S6). Based on the initial investigation of rattan genome, we had performed the identification of SNP based on transcriptomic data and the result indicated 482,692 transitions (248,810C/T and 233,882A/G transitions) and 315,263 transversions (84,024A/T, 80,905A/C, 79,469G/T and 70,865C/G transversions) (see Supplementary Table S25).

We also identified the transcriptome-based microsatellites, also called simple sequence repeats (SSRs) and developed molecular markers for assisting taxonomic study in rattan based on unigenes. Because the mono-nucleotide repeat-motif included homo-polymorphisms^27^, the repeat-motifs with 2–6 bp were selected to develop the molecular markers. We then identified 14,693 SSRs with 2–6 bp nucleotide repeats among the most abundant unigenes. The majority of SSRs had less than 24 repeat units and was dominant in 5–8 repeat units. Among the totally identified SSRs, di-nucleotide repeats (48.56%) represented the most abundant microsatellite repeat units, followed by tri-nucleotide (34.85%) and tetra-nucleotide repeats (3.02%) (see Supplementary Table S26). Moreover, compound formation was defined as more than one contiguous microsatellite locus and an intervening non-repeat sequence of less than 100 bp. Interrupted lengths were analyzed according to a length between 1 and 100 bp. The results showed that the majority of interrupted lengths were distributed from 1–10 bp and the maximum number of interrupted lengths at 1 bp accounted for 8.87% of results (see Supplementary Table S27).

The statistical results are depicted as a relative frequency based on the different microsatellite repeat motifs and length repeats (see Supplementary Table S28). For the di- and tri-nucleotide repeats, AG/CT and AT/AT were the dominant repeats relative to AC/GT and CG/CG, and AAG/CCT was the most abundant repeat, followed by CCG/CGG and AAG/CTT. Moreover, the ACT/AGT and ACG/CGT repeats had a lower frequency and only accounted for only 0.90% and 4.57%, respectively.

In addition to the usage of molecular markers, SSR variations in the 5′-UTRs may have a regulatory effect on gene expression through their influence on transcription and translation and SSR expansions in the 3′-UTRs may cause transcription slippage and produce expanded mRNA. Therefore, SSRs within genes should be subjected to stronger selective pressure than other genomic regions^28^. In this study, the SSR distribution in different regions of the unigenes indicated that 29.74%, 30.25%, and 33.07% of the sequences were mapped onto the CDS, 5′-UTR, and 3′-UTR regions, respectively (see Supplementary Table S29). The SSR motif (CCG)_6_ located in the *Djlhcb1* (KU363040) CDS region and motif (CAG)_5_ in the *DjCSE1* (KU363036) CDS region were confirmed by gene cloning and sequencing. However, the functional analysis of SSRs in genes requires a more thorough investigation and should be verified in further studies.

**Figure 5:**
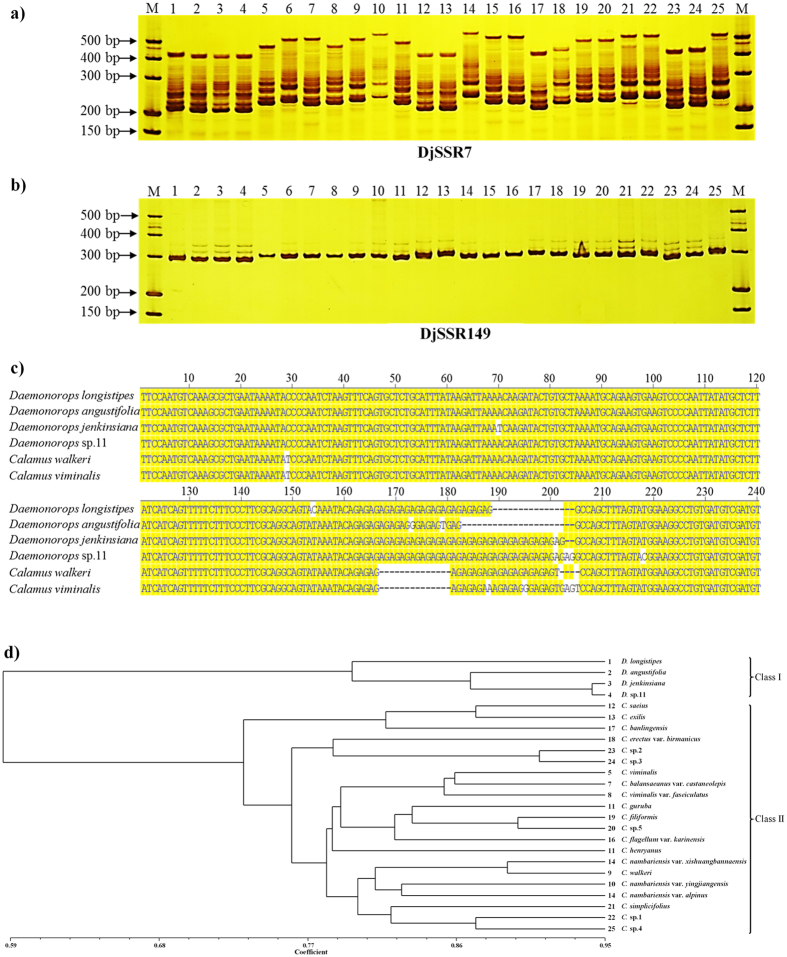
Primer pair amplification and variation analysis. (**a**) SDS-PAGE electrophoresis analysis of PCR products based on the primer pair of DjSSR7. (**b**) SDS-PAGE electrophoresis analysis of PCR products based on the primer pair of DjSSR149. No. 1–25 represents different rattan accessions, which are detailed in Supplementary Table S11. M: DNA molecular marker. (**c**) The multiple sequence alignment demonstrating the presence of a microsatellite repeat motif in 6 selected accessions based on the amplification using the primer pair of DjSSR48. Shade residues with solid bright yellow represent the consensus. (**d**) Phylogenetic tree of the 25 accessions of rattan based on microsatellite data. No. 1–25 represents different rattan accessions, which are detailed in the Supplementary Table S33.

The transcriptome-based identification of a small number of SNPs resulted in the difficult in selecting potential SNP markers. Therefore, we performed further experimental analyses of assisting taxonomic study using SSR data. Based on the batch prediction of Primer3 (see Supplementary Table S30), 9,723 PCR primers pairs of 14,693 putative SSR motifs were successfully designed. To avoid the bias and specificity in selecting SSR markers, 168 SSR markers were averagely selected from above 6 clustering DEG groups and then their primers were synthesized (see Supplementary Table S31). We then employed an experimental method to verify the identified microsatellite markers. The six rattan species from two different genera (*D. longistipes, D. angustifolia, D. jenkinsiana, D*. sp.11, *Calamus walker*, and *C. viminalis*) were utilized and amplified using the 168 primer pairs. The results demonstrated that 153 of the selected markers produced clear and stable bands at the expected size, with 127 of these markers presenting a polymorphism. The maximum number of alleles was 16, and it was observed at locus DjSSR7 (Fig. 5a), whereas the minimum number was 4, and it was detected at locus DjSSR149 (Fig. 5b). The universality and polymorphism probabilities of the different types of microsatellites are predicted in Table 1.

Furthermore, allelic length variations were detected based on differences in the microsatellite repeats. To investigate whether the PCR assay sufficiently amplified the targets, the products of the genomic DNA templates of the six species using the primer pair of DjSSR48 were subcloned into the T-easy vector and then sequenced. The results of the sequence alignment showed that locus DjSSR48 amplified alleles varying from 211 bp to 240 bp (Fig. 5c), and this result revealed a mixed allelic distribution with a variable number of repeats in the microsatellite motifs (AG)_n_ accompanied by several point mutations, such as insertion, deletion and substitution. This result confirmed that the polymorphisms of the SSR markers were relatively reliable.

In addition, the 16 primer pairs with polymorphisms were used for a polymorphism analysis among the 25 rattan accessions (see Supplementary Table S32). The 156 alleles ranged from 4 to 16 per loci and were detected by 16 SSRs, and they had an average value of 9.75. A neighbor-joining tree calculated from the microsatellite data showed that 25 accessions were clustered into 2 classes (Fig. 5d) based on genetic distance (see Supplementary Table S33). The accessions in Class I belonged to the *Daemonorops* genus, whereas the accessions in Class II belonged to the *Calamus* genus, which was consistent with the morphological classification. This result provided important data for the identification of germplasms and new evidence to support the taxonomy of Calaminae.

## Discussion

In our study, we had firstly transcriptome-based investigated the cirrus development by *de novo* transcript assembly, unigene annotation and the DEGs analysis. The development of cirri involves the elongation of the distal rachis, by which it could easily catch on to the vegetation and produce hooks to the supports in forest canopy. According to the cirrus transcriptomic data and the specific expression patterns of DEGs in *D. jenkinsiana*, we identified a number of potential genetic information that might explain the ability of the cirrus to rapidly and effectively expand in the rainforest of tropical regions. The result showed some DEGs were associated with developmental pathways, cell wall metabolism, and hook formation. For example, the genes involved in the significant pathways ‘ribosome (ko03010)’ in genetic information processing, ‘gap junction (ko04540)’ and ‘phagosome (ko04145)’ in cellular processes as well as ‘phenylpropanoid biosynthesis (ko00940)’ in metabolism were enriched in the CI *vs*. CD. In addition, the number of down-regulated genes was approximately 3-times greater that of the up-regulated genes in the HI *vs*. HD comparison, and the function of these down-regulated genes was focused on cellulose synthase, ribosomal proteins and certain transcription factors. Moreover, we found that the genes involved in cell wall metabolism were also enriched in the DGEs of two developmental stages. According to the heatmap of the expression levels (see Supplementary Fig. S7), the genes for hooks were expressed to a greater degree than those for the cirrus, which were involved in the metabolic processes of cellulose, hemicellulose, and lignin. Interestingly, highly expressed genes and significant DEGs were detected in the lignin metabolic processes of the hooks. These results were consistent with the lignification levels of the cirrus and hooks as well as the variation in the size and strength of the hooks along with cirri development^29^. Furthermore, these results provided the evidence of the underlying development and timing, which associated with hook strength and stiffness along with the development of cirri.

In addition, the cell walls are initiated through the action of a phragmoplast composed of microtubules and actin filaments, which guide the deposition of vesicles containing cell wall material to the growing cell plate^30^,^31^. It has been demonstrated that cortical microtubules and microtubule-associated proteins play an important roles in the targeted secretion of cellulose synthase complexes, the oriented deposition of cellulose microfibrils and the patterned deposition of secondary walls^32^, which would determine the feature of the wood. In this study, highly significant GO terms involved in the actin cytoskeleton and microtubules were found in the hook-specific comparative analysis, such as ‘actin cytoskeleton,’ ‘actin filament-based process,’ ‘microtubule-based process,’ and ‘microtubule associated complex.’ A large number of expressed genes related to the cytoskeleton, actin and microtubules were enriched, which may be related to the formation of hook strength. Additionally, the ‘cellular polysaccharides metabolic process’ term was detected in the cirrus-specific comparative GO enrichment analysis, which indicated that polysaccharides may be involved in the development of the cirri of *D. jenkinsiana*. Polysaccharides are a vital compound found in certain rattans, and they are known for their medicinal anticoagulant properties^33^, which is an in-depth study of direction for their exploitation.

Although rattans lack secondary growth, they develop long internodes and possess wider xylem vessels than their nonclimbing relatives^7^,^8^,^9^. Under natural conditions, the stems of rattans often reach more than 30 m in length and are attached to the surrounding vegetation through the support of relatively few cirrate leaves or flagella. Therefore, the development of cirri and flagella is very important for the climbing ability and growth patterns of rattan. Moreover, cirri and flagella are often used as morphological evidence in evolutionary and taxonomic studies^11^; however, multiple origins have been postulated for the rattan cirrus, which makes some problematic use. To improve the resolution of the phylogenetic relationships among the genera and species of Arecaceae, considerable progress had been made in the field through the use of modern molecular techniques.

Molecular markers were developed and used for the genetic diversity and phylogenetic analyses, which were helpful in the taxonomic classification of rattan. Molecular phylogenetic analyses were conducted for *Calamus* (Palmae) and related rattan genera based on the 5S nrDNA spacer sequence^34^. Moreover, simultaneous analyses of the phylogenetic relationships among the members of the subfamily Calamoideae have been performed using three independent datasets, including two molecular datasets of DNA sequences from the nuclear ribosomal internal transcribed spacer (ITS) region and the chloroplast rps16 intron^35^ and one morphological dataset^36^,^37^. Molecular evidence from Calamoideae has provided an optimal framework for developing a new understanding of character evolution and the relationships between different subfamilies^38^. However, ambiguities still persist in the members of Arecaceae. In our study, another effort was focused on the identification and development of SSR markers in *D. jenkinsiana* for molecular marker assisted taxonomy for rattan. Based on transcriptomic data, we initially identified the two kinds of biomarker (SNP and SSR). However, we ultimately developed SSR markers due to the less of candidate SNPs. Moreover, SSR markers were averagely selected from above 6 clustering DEG groups to avoid biases and specificity in selecting SSR marker. Additionally, microsatellites are one of the most powerful genetic markers in biology, and they provide a number of advantages that increase the suitability of microsatellites for genetic diversity analyses^39^, marker-assisted selection^40^, cultivar identification^41^, high-density linkage maps^42^, and QTL mapping^38^. Two single-copy nuclear genes containing microsatellite loci of the (TC)_n_ motif of intron 7 of *AG1* and the (GGC)_n_ motif of exon 1 of *PHYB* have been successfully used for palm phylogenetics^43^. In this study, 14,693 microsatellites with 2–6 bp nucleotide repeats were identified based on the unigenes of *D. jenkinsiana*. The universality and polymorphism probability were investigated using an experimental method and 16 primer pairs with polymorphisms were used in the phylogenetic analyses. The results indicated that the 25 rattan accessions were clustered into two classes that belonged to the *Daemonorops* genus and *Calamus* genus. The result was consistent with the phenotype-based taxonomy. The accessions of *D*. sp.11 and *D. jenkinsiana* were clustered closely in Class I, and the genetic distance between them was 0.095, which indicated that *D*. sp.11 might be a form of *D. jenkinsiana*. In Class II, *C*. sp.1 and *C*. sp.4 were clustered together near *C. simplicifolius*, and the genetic distance between them was 0.385, indicating that they might be different variants of *C. simplicifolius*. The genetic distance between *C*. sp.2 and *C*. sp.3 was 0.223, and they were closely clustered with *C. erectus* var. *birmanicus*, which suggested that *C*. sp.2 and *C*. sp.3 were different variants of *C. erectus*. Based on the genetic distance between *C*. sp.5 and *C. filiformis* of 0.367, *C*. sp.5 might be considered a variant of *C. filiformis*^28^.

Along with the morphological characteristics, the DEGs and microsatellite markers based on the cirrus transcriptome from the different developmental stages of *D. jenkinsiana* observed in our study will be useful for gene functions and genetic study of *D. jenkinsiana*. Therefore, the addition of molecular datasets and the exclusion of morphological data could be essential for resolving the remaining problems related to rattan taxonomy and accelerating molecular breeding programs in the future.

## Methods

### Material collection of rattan tissues

The four samples were collected in *D. jenkinsiana*. These samples were the distal cirrus without hooks at the initial stage (CI) and developed stage (CD), the third hooks from the distal end of the cirrus at the initial stage (HI) and developed stage (HD) (see Supplementary Fig. S1). All of the samples were obtained in spring from the Research Institute of Tropical Forestry of the Chinese Academy of Forestry in the city of Guangzhou, Guangdong Province, China. In addition, accession leaf samples were collected from the Xishuangbanna Tropical Botanical Gardens, Chinese Academy of Sciences, Mengla, Yunnan Province, China. The Latin names of the accessions (see Supplementary Table S32) were obtained from the Flora of China^44^ and the plant introduction and conservation database of the Xishuangbanna Tropical Botanical Gardens (http://sdb.xtbg.ac.cn/myweb/page/search.vpage).

### RNA isolation, cDNA library construction, and sequencing

Based on the manufacturer’s instructions, total RNA was isolated from selected rattan tissue using TRIzol Reagent Solution (Invitrogen, Carlsbad, CA, USA). The purity and concentration were determined with a NanoDrop 2000 spectrophotometer. Reverse transcription was conducted with a Reverse Transcription System (Promega, USA). The extracted RNA was treated with RNase-free DNase I for 30 min at 37 °C to remove the residual DNA as previously described^45^,^46^, and the pooled libraries were then sequenced using the Illumina HiSeq 2500 platform (Illumina) at Novogene Bioinformatics Technology Co., Ltd, Beijing, China. Finally, the detailed information about RNA libraries was provided in Supplementary Table S1.

### Bioinformatics analysis for transcriptomic data

The transcriptomic data were filtered and cleaned by removing the adaptor sequences and low-quality reads using Trimmomatic (version 0.36)^47^ with default parameters. The filtered reads of all samples were randomly clipped into 25-mers, which were subsequently assembled using Trinity (version 2.0.6)^48^ with default parameters. We then removed false positive transcripts which had short length and low expression level, with the following cut-off: the number of transcriptomic reads mapping into assembling transcripts in all samples, termed the total reads count (TRC), was less than 40. Finally, we calculated TRC for each transcript because a gene might find various transcripts. For the gene which had more than one transcript, the transcript with the highest TRC was considered as a unigene. All unigenes, termed as the unigenes of the rattan, were used for further functional analysis. After the assembling and annotation, the completeness of the unigenes according to conserved orthologous content was evaluated by BUSCO (version 2.0)^49^ with its plant reference dataset (embryophyta_odb9).

In functional analysis, all unigenes (61,569 unigenes) were aligned against the following databases: non-redundant NCBI protein sequence (dataset v2015.08.13), UniProtKB/SwissProt (dataset v2015.09), GO (dataset v2013-10-30), COG (dataset v2003), KEGG (dataset from KEGG Automatic Annotation Server v2.1), InterPro (dataset v.53), and CDD (dataset v3.14). The above BLAST searches were totally conducted with a cut-off e-value of <10^−5^ 50,51. Moreover, the fragments per kilobase per million (FPKM) for each gene was calculated by the script program in Trinity and the two software programs: RSEM (version 1.2.30)^52^ for the expression estimated method and bowtie (version 0.12.3)^53^ for the alignment method. We calculated the number of clean tags for every gene and compared the expression levels to identify the significant DEGs using edgeR package (version 3.16.5)^54^ with the following cut-off values: adjusted *p*-value < 0.001, | log_2_(fold change) |>2 and the method of TTM (Trimmed Mean of M-values) for the normalization and comparative analysis. The clustering analysis of DEGs is based on the distance between two genes. We have used the Euclidean methods for calculating the distance and then used complete linkage method for clustering. For GO annotation, the two software of BLAST2GO (version 2.5)^50^ and InterProScan (version 5.10-50.0)^55^ were capability to obtain GO information based on alignment analysis. The two software were used with a cut-off e-value <1e^−5^ in our study. We ultimately obtained the GO result after integrated with the results from two sources. For the enrichment analysis, totally unigenes were considered as the background. The software of Ontologizer (version 2.0)^51^ was used with the method of “Parent-Child-Union” and *p*-values were adjusted by the Bonferroni’s method. The significant enriched GO terms were identified with *p*-value < 0.01. Furthermore, the potential SSR and SNP markers were predicted to identify potential new molecular markers based on the transcriptomic data. The microsatellites in the unigenes were identified using MISA (version 1.0 and http://pgrc.ipk-gatersleben.de/misa/) with default parameters. The minimum repeat unit was defined as 6 for the dinucleotides and 5 for the tri-, tetra-, penta-, and hexa-nucleotides. The microsatellite position and different microsatellites repeat types and lengths were analyzed with a bespoke program written using MISA files. All of the common and unique SNP sites among all of the samples were predicted using mpileup, as one of scripts in SAMtools (version 1.2 and http://github.com/samtools/).

### Verification of the sequenced and assembled data quality

Relatively accurate sequenced and assembled data are required for further analyses, such as sequence annotation, variation detection and expression analyses. Therefore, twelve unigenes were randomly selected, and the primer pairs for the RT-PCR amplification were designed and synthesized. The leaf cDNA samples of *D. jenkinsiana* were used as a template for the RT-PCR, and the PCR products at the expected product size were subcloned into a T-easy vector and then sequenced. The sequence alignment analysis was performed with DNASTAR (version 7.10 and http://www.dnastar.com) with default parameters.

### Validation of DEGs in different samples

The total RNAs were isolated from the four independently collected tissues respectively, which were in the same developmental stage as those used for the RNA-Seq analysis. First-strand of cDNA was synthesized by Reverse Transcription System (Promega, USA). For each 20 μl reaction 1 μg of total RNA was used, the synthesis was carried out at the 42 °C for 15 min and the final cDNA product was diluted 5-fold prior to use. Thirty-seven DEGs were selected randomly and primer pairs for quantitative analysis were designed (see Supplementary Table S14). Besides, all primers showed a clear specific melting peak by real-time melting curve analysis, which agreed with the results of agarose gel electrophoresis for specific PCR product respectively, were used for further analysis. Quantitative real time PCR (qRT-PCR) assay was performed on qTOWER2.2 (Analytik Jena, Germany) with Roche LightCycler^®^480 SYBR Green 1 Master kit, according to the procedure of 95 °C for 5 min; 95 °C for 10 s, 58–64 °C for 10 s, 40 cycles. The reaction volume was 10 μL containing 5.0 μL of 2 × SYBR Green 1 Master, 0.8 μL cDNA, 0.2 μL of forward primer and reverse primer (5 μmol·L^−1^, each) and 3.8 μL ddH_2_O. Calibration curves were obtained by amplification using a 10-fold serial dilution of each cDNA sample, the correlation coefficients (R^2^) of curves were also ranged from 0.990 to 0.999 and the PCR efficiency values were between 0.95 and 1.05 (see Supplementary Table S18). For each condition, the qRT-PCR experiments were performed in biological triplicate with three technical replicates. Gene expression was calculated with double delta Ct method using γ-tubulin gene (Assembly name: TR97697_c2_g9_i2) as the reference gene from three candidate genes (γ-tubulin, actin, and ubiquitin). The detailed information was provided in Supplementary Table S34.

### Primer design and microsatellite marker validation

Based on the assembled unigene sequences containing the microsatellite motif, the primer pairs for the flanking sequences of each unique microsatellite were designed automatically using Primer3.0 (version 2.3.6 and see Supplementary Table S30 for whole parameters) and then synthesized (see Supplementary Table S21). The genomic DNA of the leaf samples was extracted using the CTAB method, PCR amplification was conducted, and the PCR products were separated using the PAGE method according to previous studies^27^. Six samples (*Daemonorops longistipes, D. angustifolia, D. jenkinsiana, D*. sp.11, *Calamus walker*, and *C. viminalis*) were selected to validate the suitability of the microsatellite loci. Moreover, 25 accessions belonging to two genera, including four from *Daemonorops* and twenty-one from *Calamus* (see Supplementary Table S22), were used for the microsatellite marker validation. A neighbor-joining cluster analysis was performed to demonstrate the relationships based on the similarity matrix for the 25 rattan accessions using NTSYSpc (version 2.10 and http://ntsyspc.software.informer.com/2.10/) with default parameters. The parameters of the neighbor-joining cluster analysis were as follows: Method: WEIGHTED, Maximum no. tied trees: 25, Tie tolerance: 0.000000000, Rooting method: MIDPOINT and in case of ties: FIND.

### Variation analysis of microsatellite markers

Three primer pairs were selected to detect the microsatellite variations in the different rattan species. The genomic DNA samples of *D. longistipes, D. angustifolia, D. jenkinsiana, D*. sp.11, *C. walkeri*, and *C. viminalis* were used as the templates for the PCR assay, and the PCR products were subcloned into a T-easy vector and then sequenced. The sequence alignment analysis was conducted using DNASTAR.

### DNA library preparation, genome sequencing and genome survey

We constructed the library, sequenced and performed the investigation of genome survey. The samples were consistent with the transcriptomic sequencing of rattan in this study. The total DNA from rattan leaves was isolated and extracted using DNeasy Plant Mini kit (Qiagen) on the basis of the manufacturer’s instructions. Genomic DNA was purified based on the protocol for the isolation of high-molecular-weight nuclear DNA. Moreover, according to the standard Illumina protocol, the 250 base pairs (bp) paired-end library with insert size of ~450 bp was constructed from randomly fragmented genomic DNA. For the preprocessing, the low-quality reads (the percentage >40% and Q < 13) were filtered using NGS QC Toolkit (version 2.3.3)^56^ with default parameters. Then, we have used FindErorrs, one of the scripts in ALLPATHS-LG (version r52488)^57^, to correct the filtered reads. Thus, we have predicted the genome size, identified repeat sequence (repeat time >1 and length >24 bp), detected SNP in genome and kmer coverage of 42× based on ALLPATHS-LG and the script of Kmer, one of the scripts in GCE (version 1.0.0 and ftp://ftp.genomics.org.cn/pub/gce/). Lastly, *De novo* assembling was performed using DISCOVAR *de novo* (version r52488)^58^ with default parameters. Additionally, the above DNA library preparation and sequencing on Illumina Hiseq-4000 sequencing platform were also performed at Novogene Bioinformatics Technology Co., Ltd, Beijing, China. Finally, the detailed information about DNA libraries was provided (see Supplementary Table S23).

### The accession for high-throughput data

All of the raw sequence data were uploaded to the NCBI Sequence Read Archive (accession numbers: SRR3089417, SRR3089429, SRR3089432, SRR3089433, SRR3089434, SRR3089435, SRR3089436, and SRR3089437).

## Additional Information

**How to cite this article**: Zhao, H. *et al*. Transcriptome-based investigation of cirrus development and identifying microsatellite markers in rattan (*Daemonorops jenkinsiana*). *Sci. Rep.* **7**, 46107; doi: 10.1038/srep46107 (2017).

**Publisher's note:** Springer Nature remains neutral with regard to jurisdictional claims in published maps and institutional affiliations.

## Supplementary Material

Supplementary Data

Supplementary File

Supplementary Table S6

Supplementary Table S7 Protein

Supplementary Table S7 Nucleotide

## Figures and Tables

**Table 1:** Universal and polymorphic potential of the 168 microsatellite markers.

Type of SSR	Number of SSR	Universality		Polymorphism	
		Number	Probability (%)	Number	Probability (%)
Di-nucleotide	91	83	91.2	74	89.2
Tri-nucleotide	40	38	95.0	35	92.1
Tetra-nucleotide	12	11	91.7	11	100
Penta-nucleotide	5	4	80.0	4	100
Hexa-nucleotide	3	3	100	3	100
Compound	17	14	82.4	14	100
Total	168	153	—	127	—

## References

[b1] Johnson D. V. Rattan Glossary: And Compendium Glossary with Emphasis on Africa. Vol.1 Ch.2, 61–62 (Food and Agriculture Organization, 2004).

[b2] Raj H. Yadav S. & Bisht N. S. Current status, issues and conservation strategies for Rattans of North-East India. Trop Plant Res 2, 1–7 (2014).

[b3] Noor N. S., Hamzah K. A. & Mohd W. R. Considerations in rattan inventory practices in the tropics. 9–12 (International Network for Bamboo and Rattan, 1999).

[b4] Zhao X., Huang S., Xian G. & Li R. Analysis and evaluation of nutritional components in *Daemonorops margaritae* Shoots. Nonwood Forest Research 25, 46–48 (2007).

[b5] Jiang Z., Lü W., Fei B., Ren H., Wu Y. & Wang Z. Anatomical characteristics of three commercial rattan canes in south China. Scientia Silvae Sinicae 43, 121–126 (2007).

[b6] Lü W., Jiang Z. & Wu Y. Basic components and chemical properties of the cane of *Daemonorops margaritae*. Scientia Silvae Sinicae 45, 96–100 (2009).

[b7] Tomlinson P. B., Fisher J. B., Spangler R. E. & Richer R. A. Stem vascular architecture in the rattan palm *Calamus* (Arecaceae-Calamoideae-Calaminae). Am J Bot 88, 797–809 (2001). 11353705

[b8] Tomlinson P. B. & Spangler R. Developmental features of the discontinuous stem vascular system in the rattan palm *Calamus* (Arecaceae-Calamoideae-Calamineae). Am J Bot 89, 1128–1141 (2002). 21665713 10.3732/ajb.89.7.1128

[b9] Tomlinson P. B. & Zimmermann M. H. Stem vascular architecture in the American climbing palm *Desmoncus* (Arecaceae-Calamoideae-Calamineae). Botl J Lin Soc 142, 243–254 (2003).

[b10] Isnard S. & Rowe N. P. The climbing habit in palms: Biomechanics of the cirrus and flagellum. Am J Bot 95, 1538–1547 (2008). 21628161 10.3732/ajb.0700005

[b11] Baker W. J., Dransfield J. & Hedderson T. A. Phylogeny, character evolution, and a new classification of the calamoid palms. Systematic Botany 25, 297–322 (2000).

[b12] Dransfield J. A synopsis of the genus *Korthalsia* (Palmae: Lepidocaryoideae). Kew Bulletin, 163–194 (1981).

[b13] Pajoro A. . Dynamics of chromatin accessibility and gene regulation by MADS-domain transcription factors in flower development. Genome Biol 15, R41, doi: 10.1186/gb-2014-15-3-r41 (2014). 24581456 PMC4054849

[b14] Galata M., Sarker L. S. & Mahmoud S. S. Transcriptome profiling, and cloning and characterization of the main monoterpene synthases of Coriandrum sativum L. Phytochemistry 102, 64–73, doi: 10.1016/j.phytochem.2014.02.016 (2014). 24636455

[b15] Borden J. R. & Papoutsakis E. T. Dynamics of genomic-library enrichment and identification of solvent tolerance genes for Clostridium acetobutylicum. Appl Environ Microbiol 73, 3061–3068, doi: 10.1128/AEM.02296-06 (2007). 17337545 PMC1892849

[b16] Maretty L., Sibbesen J. A. & Krogh A. Bayesian transcriptome assembly. Genome Biol 15, 501, doi: 10.1186/s13059-014-0501-4 (2014). 25367074 PMC4397945

[b17] Wan Y. . Transcriptome analysis of grain development in hexaploid wheat. BMC Genomics 9, 121, doi: 10.1186/1471-2164-9-121 (2008). 18325108 PMC2292175

[b18] Zhu Q. H. . Characterization of the defense transcriptome responsive to Fusarium oxysporum-infection in Arabidopsis using RNA-seq. Gene 512, 259–266, doi: 10.1016/j.gene.2012.10.036 (2013). 23107761

[b19] Wu Z. & Wu H. Experimental Design and Power Calculation for RNA-seq Experiments. Methods Mol Biol 1418, 379–390, doi: 10.1007/978-1-4939-3578-9_18 (2016). 27008024

[b20] O’Leary N. A. . Reference sequence (RefSeq) database at NCBI: current status, taxonomic expansion, and functional annotation. Nucleic Acids Res, doi: 10.1093/nar/gkv1189 (2015). PMC4702849 26553804

[b21] The UniProt, C. UniProt: the universal protein knowledgebase. Nucleic Acids Res 45, D158–D169, doi: 10.1093/nar/gkw1099 (2017). 27899622 PMC5210571

[b22] Galperin M. Y., Makarova K. S., Wolf Y. I. & Koonin E. V. Expanded microbial genome coverage and improved protein family annotation in the COG database. Nucleic Acids Res 43, D261–269, doi: 10.1093/nar/gku1223 (2015). 25428365 PMC4383993

[b23] Gene Ontology C. Gene Ontology Consortium: going forward. Nucleic Acids Res 43, D1049–1056, doi: 10.1093/nar/gku1179 (2015). 25428369 PMC4383973

[b24] Kanehisa M., Furumichi M., Tanabe M., Sato Y. & Morishima K. KEGG: new perspectives on genomes, pathways, diseases and drugs. Nucleic Acids Res 45, D353–D361, doi: 10.1093/nar/gkw1092 (2017). 27899662 PMC5210567

[b25] Marchler-Bauer A. . CDD/SPARCLE: functional classification of proteins via subfamily domain architectures. Nucleic Acids Res 45, D200–D203, doi: 10.1093/nar/gkw1129 (2017). 27899674 PMC5210587

[b26] Finn R. D. . InterPro in 2017-beyond protein family and domain annotations. Nucleic Acids Res 45, D190–D199, doi: 10.1093/nar/gkw1107 (2017). 27899635 PMC5210578

[b27] Zhao H. . Developing genome-wide microsatellite markers of bamboo and their applications on molecular marker assisted taxonomy for accessions in the genus Phyllostachys. Sci Rep 5, 8018, doi: 10.1038/srep08018 (2015). 25620112 PMC4306134

[b28] Li Y.-C., Korol A. B., Fahima T. & Nevo E. Microsatellites within genes: structure, function, and evolution. Mol Biol Evol 21, 991–1007 (2004). 14963101 10.1093/molbev/msh073

[b29] Isnard S. & Rowe N. P. The climbing habit in palms: biomechanics of the cirrus and flagellum. Am J Bot 95, 1538–1547 (2008). 21628161 10.3732/ajb.0700005

[b30] Gunning B. The cytokinetic apparatus: Its development and apatial regulation. In The Cytoskeleton in Plant Growth and Development, Lloyd C. W. ed. (London/New York: Academic Press) 229–292 (1982).

[b31] Staehelin L. A. & Hepler P. K. Cytokinesis in higher plants. Cell 84, 821–824 (1996). 8601305 10.1016/s0092-8674(00)81060-0

[b32] Zhong R. & Ye Z. H. Secondary cell walls: biosynthesis, patterned deposition and transcriptional regulation. Plant Cell Physiol 56, 195–214, doi: 10.1093/pcp/pcu140 (2015). 25294860

[b33] Gibbs A., Green C. & Doctor V. M. Isolation and anticoagulant properties of polysaccharides of Typha Augustata and Daemonorops species. Thromb Res 32, 97–108 (1983). 6658717 10.1016/0049-3848(83)90021-x

[b34] Baker W. J., Hedderson T. A. & Dransfield J. Molecular phylogenetics of Calamus (Palmae) and related rattan genera based on 5S nrDNA spacer sequence data. Mol Phylogenet Evol 14, 218–231, doi: 10.1006/mpev.1999.0697 (2000). 10679156

[b35] Baker W. J., Hedderson T. A. & Dransfield J. Molecular phylogenetics of subfamily Calamoideae (Palmae) based on nrDNA ITS and cpDNA rps16 intron sequence data. Mol Phylogenet Evol 14, 195–217, doi: 10.1006/mpev.1999.0696 (2000). 10679155

[b36] Baker W. J., Asmussen C. B., Barrow S. C., Dransfield J. & Hedderson T. A. A phylogenetic study of the palm family (Palmae) based on chloroplast DNA sequences from thetrnL—trnF region. Plant Syst Evol 219, 111–126 (1999).

[b37] Baker W., Dransfield J., Harley M. & Bruneau A. Morphology and cladistic analysis of subfamily Calamoideae (Palmae). Mem. New York Bot. Gard 83, 307–324 (1999).

[b38] Alapetite E., Baker W. J. & Nadot S. Evolution of stamen number in Ptychospermatinae (Arecaceae): Insights from a new molecular phylogeny of the subtribe. Mol Phylogenet Evol 76, 227–240 (2014). 24632147 10.1016/j.ympev.2014.02.026

[b39] Moretzsohn M. C. . Genetic diversity of peanut (Arachis hypogaea L.) and its wild relatives based on the analysis of hypervariable regions of the genome. BMC Plant Biol. 4, 11 (2004). 15253775 10.1186/1471-2229-4-11 PMC491793

[b40] Sui C., Wei J., Chen S., Chen H. & Yang C. Development of genomic SSR and potential EST-SSR markers in Bupleurum chinense DC. African Journal of Biotechnology 8 (2009).

[b41] Ercisli S., Ipek A. & Barut E. SSR marker-based DNA fingerprinting and cultivar identification of olives (Olea europaea). Biochem Genet 49, 555–561, doi: 10.1007/s10528-011-9430-z (2011). 21476017

[b42] Sugita T. . Development of simple sequence repeat markers and construction of a high-density linkage map of Capsicum annuum. Mol Breeding 31, 909–920 (2013).

[b43] Ludena B. . Phylogenetic utility of the nuclear genes AGAMOUS 1 and PHYTOCHROME B in palms (Arecaceae): an example within Bactridinae. Ann Bot 108, 1433–1444, doi: 10.1093/aob/mcr191 (2011). 21828068 PMC3219496

[b44] Wu Zhenyi & Peter Raven. Flora of China Editorial Committee. Flora of China. Vol. 22 (Science Press, 2006).

[b45] Zhao H. . Transcriptome and comparative gene expression analysis of *Phyllostachys edulis* in response to high light. BMC Plant Biol 16, 34, doi: 10.1186/s12870-016-0720-9 (2016). 26822690 PMC4730629

[b46] Zhao H. e. a. Comprehensive analysis of multi-tissue transcriptome data and the genome-wide investigation of GRAS family in *Phyllostachys edulis*. Sci. Rep 6, 27640, doi: 10.1038/srep27640 (2016). 27325361 PMC4914925

[b47] Bolger A. M., Lohse M. & Usadel B. Trimmomatic: a flexible trimmer for Illumina sequence data. Bioinformatics 30, 2114–2120, doi: 10.1093/bioinformatics/btu170 (2014). 24695404 PMC4103590

[b48] Haas B. J. . De novo transcript sequence reconstruction from RNA-seq using the Trinity platform for reference generation and analysis. Nat Protoc 8, 1494–1512, doi: 10.1038/nprot.2013.084 (2013). 23845962 PMC3875132

[b49] Simao F. A., Waterhouse R. M., Ioannidis P., Kriventseva E. V. & Zdobnov E. M. BUSCO: assessing genome assembly and annotation completeness with single-copy orthologs. Bioinformatics 31, 3210–3212, doi: 10.1093/bioinformatics/btv351 (2015). 26059717

[b50] Conesa A. & Gotz S. Blast2GO: A comprehensive suite for functional analysis in plant genomics. Int J Plant Genomics 2008, 619832, doi: 10.1155/2008/619832 (2008). 18483572 PMC2375974

[b51] Bauer S., Grossmann S., Vingron M. & Robinson P. N. Ontologizer 2.0–a multifunctional tool for GO term enrichment analysis and data exploration. Bioinformatics 24, 1650–1651, doi: 10.1093/bioinformatics/btn250 (2008). 18511468

[b52] Langmead B. Aligning short sequencing reads with Bowtie. Curr Protoc Bioinformatics Chapter 11, Unit 11 17, doi: 10.1002/0471250953.bi1107s32 (2010). PMC3010897 21154709

[b53] Li B. & Dewey C. N. RSEM: accurate transcript quantification from RNA-Seq data with or without a reference genome. BMC Bioinformatics 12, 323, doi: 10.1186/1471-2105-12-323 (2011). 21816040 PMC3163565

[b54] Robinson M. D., McCarthy D. J. & Smyth G. K. edgeR: a Bioconductor package for differential expression analysis of digital gene expression data. Bioinformatics 26, 139–140, doi: 10.1093/bioinformatics/btp616 (2010). 19910308 PMC2796818

[b55] Jones P. . InterProScan 5: genome-scale protein function classification. Bioinformatics 30, 1236–1240, doi: 10.1093/bioinformatics/btu031 (2014). 24451626 PMC3998142

[b56] Patel R. K. & Jain M. NGS QC Toolkit: a toolkit for quality control of next generation sequencing data. PLoS One 7, e30619, doi: 10.1371/journal.pone.0030619 (2012). 22312429 PMC3270013

[b57] Maccallum I. . ALLPATHS 2: small genomes assembled accurately and with high continuity from short paired reads. Genome Biol 10, R103, doi: 10.1186/gb-2009-10-10-r103 (2009). 19796385 PMC2784318

[b58] Weisenfeld N. I. . Comprehensive variation discovery in single human genomes. Nat Genet 46, 1350–1355, doi: 10.1038/ng.3121 (2014). 25326702 PMC4244235

