# Supplementary material for: Transcriptome-based investigation of cirrus development and identifying microsatellite markers in rattan (Daemonorops jenkinsiana)
Source: Sci Rep. 2017 Apr 6;7:46107. doi: 10.1038/srep46107 (PMC5382692; doi:10.1038/srep46107)
Supplement: Supplementary Data [file srep46107-s1.doc]

***Supplementary information***

**Transcriptome-based investigation of cirrus development and** **identifying microsatellite markers in** **rattan (*Daemonorops jenkinsiana*)**

**Hansheng Zhao^1,#,+^, Huayu Sun^1,#^, Lichao Li^1^, Yongfeng Lou^1^, Rongsheng Li^2^, Lianghua Qi^1^, and Zhimin Gao^1,+^**

^1^ State Forestry Administration Key Open Laboratory on the Science and Technology of Bamboo and Rattan, International Center for Bamboo and Rattan, Beijing 100102, China.

^2^ Research Institute of Tropical Forestry, Chinese Academy of Forestry, Guangzhou, 510000, China.

^+^ Co-corresponding author. zhaohansheng@icbr.ac.cn, and gaozhimin@icbr.ac.cn

^#^ These authors contributed equally to this work.

*Index*

[**Supplementary Figure S1: Samples collected from the different developmental stages.** 4](#__RefHeading___Toc473652287)

[**Supplementary Figure S2. The distribution of the reads randomness in the three biological repeated.** 5](#__RefHeading___Toc473652288)

[**Supplementary Figure S3: Summary of the gene expression levels and the differentially expression genes.** 6](#__RefHeading___Toc473652289)

[**Supplementary Figure S4: Validation of RNA-Seq data by qRT-PCR.** 8](#__RefHeading___Toc473652290)

[**Supplementary Figure S5: The heatmap of clustering analysis.** 10](#__RefHeading___Toc473652291)

[**Supplementary Figure S6: The distribution of 17Kmer to estimate the genome size of *D. jenkinsiana*.** 11](#__RefHeading___Toc473652292)

[**Supplementary Figure S7: Heat map of the differentially expressed genes involved in the metabolic processes of cellulose, hemicellulose, and lignin.** 12](#__RefHeading___Toc473652293)

[**Supplementary Table S1: the statistics for transcriptomic libraries in rattan** 13](#__RefHeading___Toc473652294)

[**Supplementary Table S2: Summary of the output statistics from selected tissues in *D. jenkinsiana*** 14](#__RefHeading___Toc473652295)

[**Supplementary Table S3: The summary of the DEGs in two datasets** 15](#__RefHeading___Toc473652296)

[**Supplementary Table S4: The comparative analysis of the two biological replicates and the three ones** 15](#__RefHeading___Toc473652297)

[**Supplementary Table S5: Summary of *de novo* sequence assembly by trinity software** 16](#__RefHeading___Toc473652298)

[**Supplementary Table S6: Summary of trinity genes and unigenes** 17](#__RefHeading___Toc473652299)

[**Supplementary Table S7: The all sequence of unigenes (both protein and nucleotide sequences)** 18](#__RefHeading___Toc473652300)

[**Supplementary Table S8: The completeness of unigenes by BUSCO** 19](#__RefHeading___Toc473652301)

[**Supplementary Table S9: List of 10 genes isolated and 2 genes failed to isolate by RT-PCR** 20](#__RefHeading___Toc473652302)

[**Supplementary Table S10: The result of Gene Ontology classification** 21](#__RefHeading___Toc473652303)

[**Supplementary Table S11: The result of COG annotation of putative proteins** 23](#__RefHeading___Toc473652304)

[**Supplementary Table S12: The KEGG pathway enrichment analysis** 24](#__RefHeading___Toc473652305)

[**Supplementary Table S13: The result of species distribution via the comparative analysis of homologous genes** 28](#__RefHeading___Toc473652306)

[**Supplementary Table S14: The list of the up-regulated genes in CI *vs.* CD** 30](#__RefHeading___Toc473652307)

[**Supplementary Table S15: The list of the down-regulated genes in CI *vs.* CD** 31](#__RefHeading___Toc473652308)

[**Supplementary Table S16: The list of the up-regulated genes in HI *vs.* HD** 32](#__RefHeading___Toc473652309)

[**Supplementary Table S17: The list of the down-regulated genes in HI *vs.* HD** 33](#__RefHeading___Toc473652310)

[**Supplementary Table S18: 37 primer pairs used for qRT-PCR expression analysis for validating the expressed value** 34](#__RefHeading___Toc473652311)

[**Supplementary Table S19: The list of the significant GO terms** 36](#__RefHeading___Toc473652312)

[**Supplementary Table S20: The GO terms enrichment analysis of the down-regulated genes in CI *vs.* CD** 37](#__RefHeading___Toc473652313)

[**Supplementary Table S21: The GO terms enrichment analysis of the up-regulated genes in HI *vs.* HD** 38](#__RefHeading___Toc473652314)

[**Supplementary Table S22: The GO terms enrichment analysis of the down-regulated genes in HI *vs.* HD** 39](#__RefHeading___Toc473652315)

[**Supplementary Table S23: The information on DNA PCR-free library** 42](#__RefHeading___Toc473652316)

[**Supplementary Table S24: The summary of genome survey for *D. jenkinsiana*** 43](#__RefHeading___Toc473652317)

[**Supplementary Table S25: Summary of single nucleotide polymorphisms** 44](#__RefHeading___Toc473652318)

[**Supplementary Table S26: Summary of microsatellites in *Daemonorops jenkinsiana*** 45](#__RefHeading___Toc473652319)

[**Supplementary Table S27: The distribution of interrupted length** 46](#__RefHeading___Toc473652320)

[**Supplementary Table S28: Frequency of di- to hexa-nucleotide repeat motifs** 47](#__RefHeading___Toc473652321)

[**Supplementary Table S29: The SSR distribution in different regions of unigenes** 49](#__RefHeading___Toc473652322)

[**Supplementary Table S30: The whole parameters of Primer3** 50](#__RefHeading___Toc473652323)

[**Supplementary Table S31: List of 168 primer pairs used in the study for amplification** 53](#__RefHeading___Toc473652324)

[**Supplementary Table S32: The list of 25 accessions used in this study** 54](#__RefHeading___Toc473652325)

[**Supplementary Table S33: The genetic distance between the 25 accessions used in this study** 55](#__RefHeading___Toc473652326)

[Supplementary Table S34. The expression value of candidate reference genes in 8 samples 56](#__RefHeading___Toc473652327)


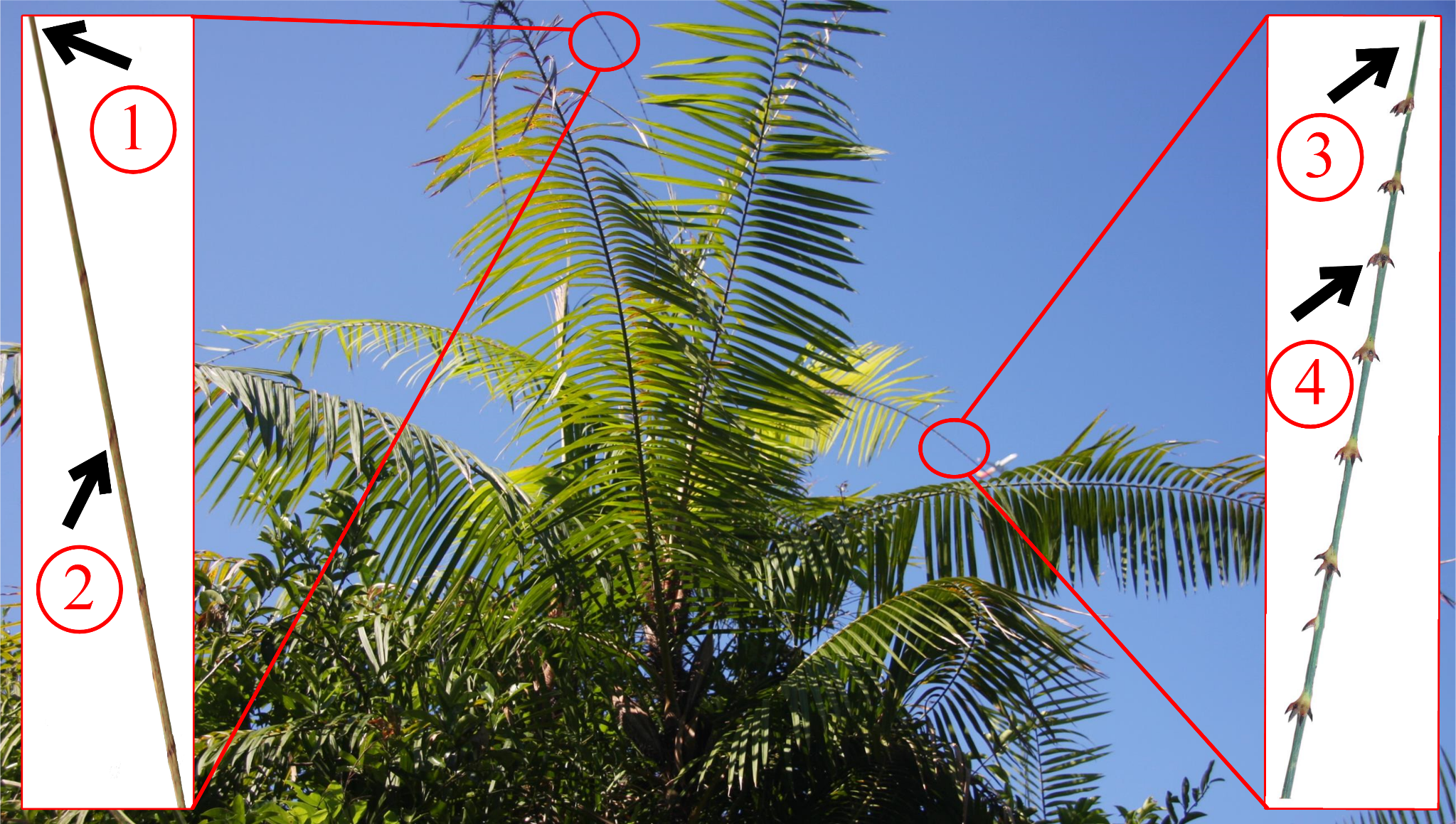
Supplementary Figure S1: Samples collected from the different developmental stages.

1: CI sample, 2: HI sample, 3: CD sample, 4: HD sample


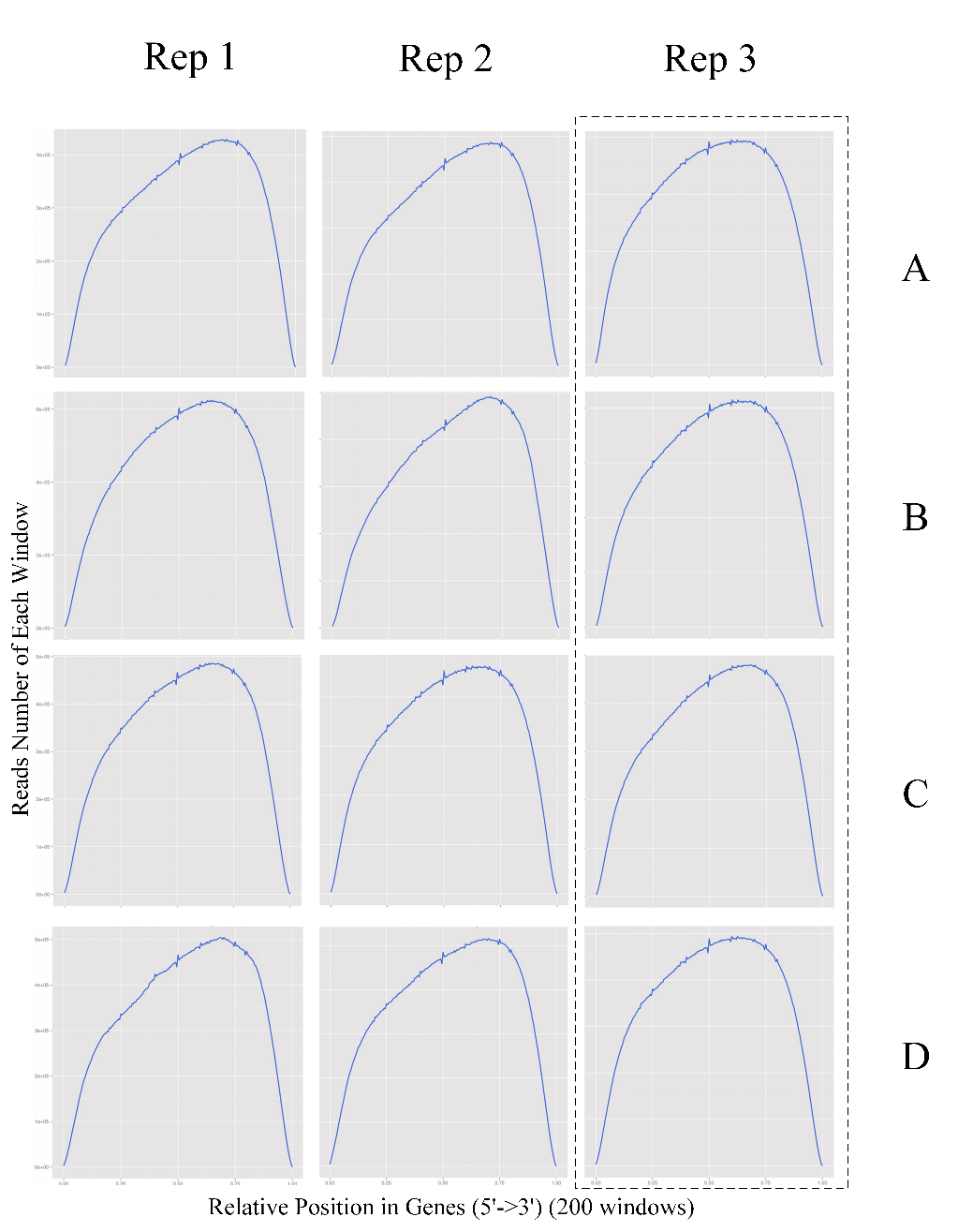


Supplementary Figure S2. The distribution of the reads randomness in the three biological repeated.

Both the Rep1 and Rep2 were produced by the sequencing. The Rep3 in dotted line was produced by the random sampling based on the sequencing data. A: CI sample; B: CD sample; C: HI sample; D: HD sample.


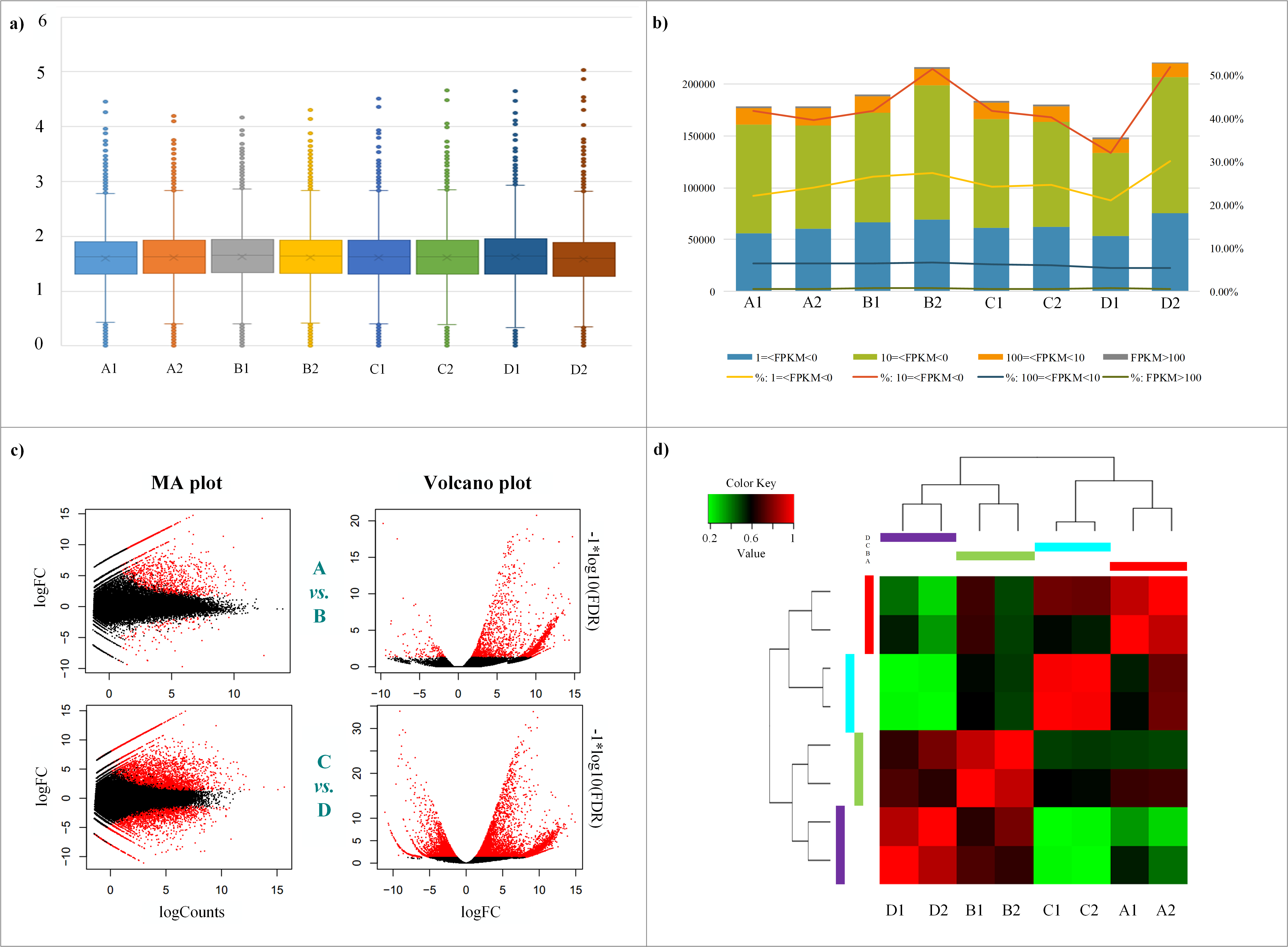


**Supplementary Figure S3: Summary of the gene expression levels and the differentially expression genes.**

(a) Boxplot results of the gene expression levels. The expressed level equals log10 (FPKM+1). The outlier data were displayed and the cross mark (X) represented the mean value.

(b) Distribution level of the expressed genes.

(c) MA and volcano plots of the significant expressed genes for a pair of samples.

(d) Clusters of FPKM correlations in the eight samples.

A: CI sample, B: CD sample, C: HI sample and D: HD sample. 1: replicate 1; and 2: replicate 2.


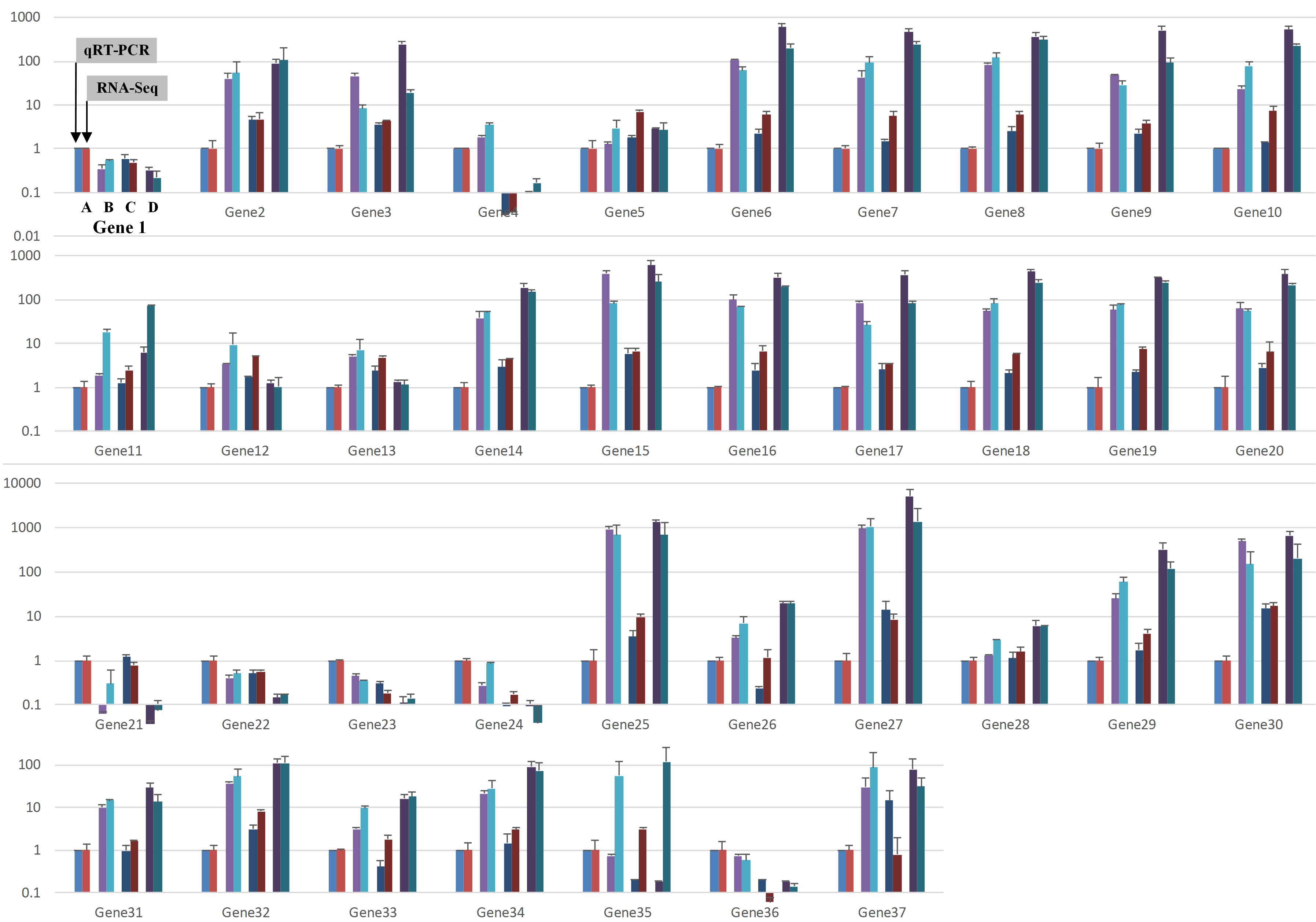


**Supplementary Figure S4: Validation of RNA-Seq data by qRT-PCR.**

Comparison of relative expression of 37 selected genes based on RNA-Seq data and qRT-PCR data. A histogram of gene expression combined RNA-Seq data with qRT-PCR data. X-axes represented 37 selected genes randomly. Y-axes represented relative expression. A: CI sample (Blue box represented qRT-PCR, red box represented RNA-Seq); B: CD sample (Purple box represented qRT-PCR, light blue box represented RNA-Seq); C: HI sample (Dark blue box represented qRT-PCR, dark red box represented RNA-Seq); D: HD sample (Dark purple box represented qRT-PCR, light blue-green box blue box represented RNA-Seq). Error bars indicate standard deviation in both RNA-Seq data and qRT-PCR data.


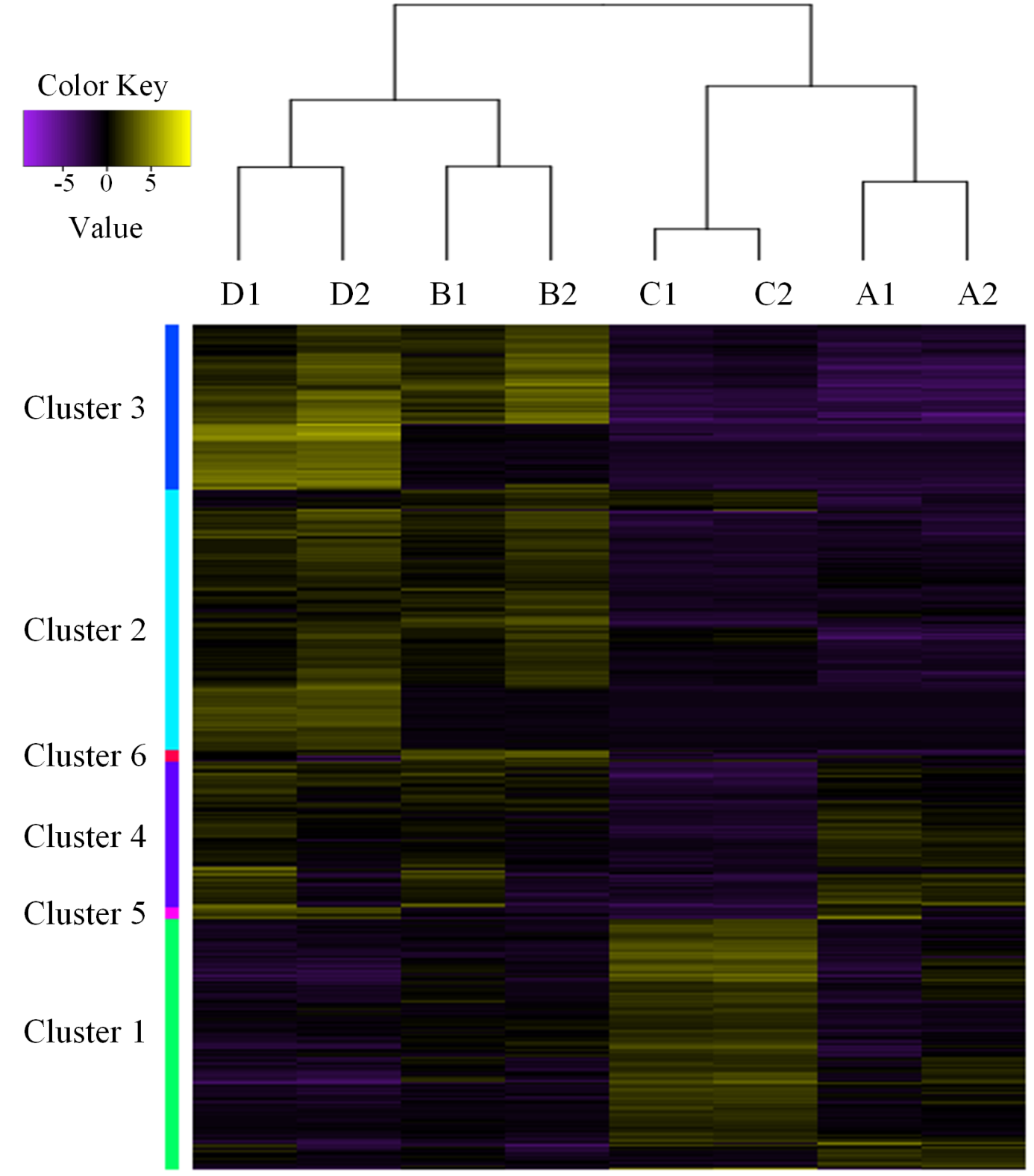


Supplementary Figure S5: The heatmap of clustering analysis.

A: CI sample, B: CD sample, C: HI sample, D: HD sample, 1: replicate 1, and 2: replicate 2.


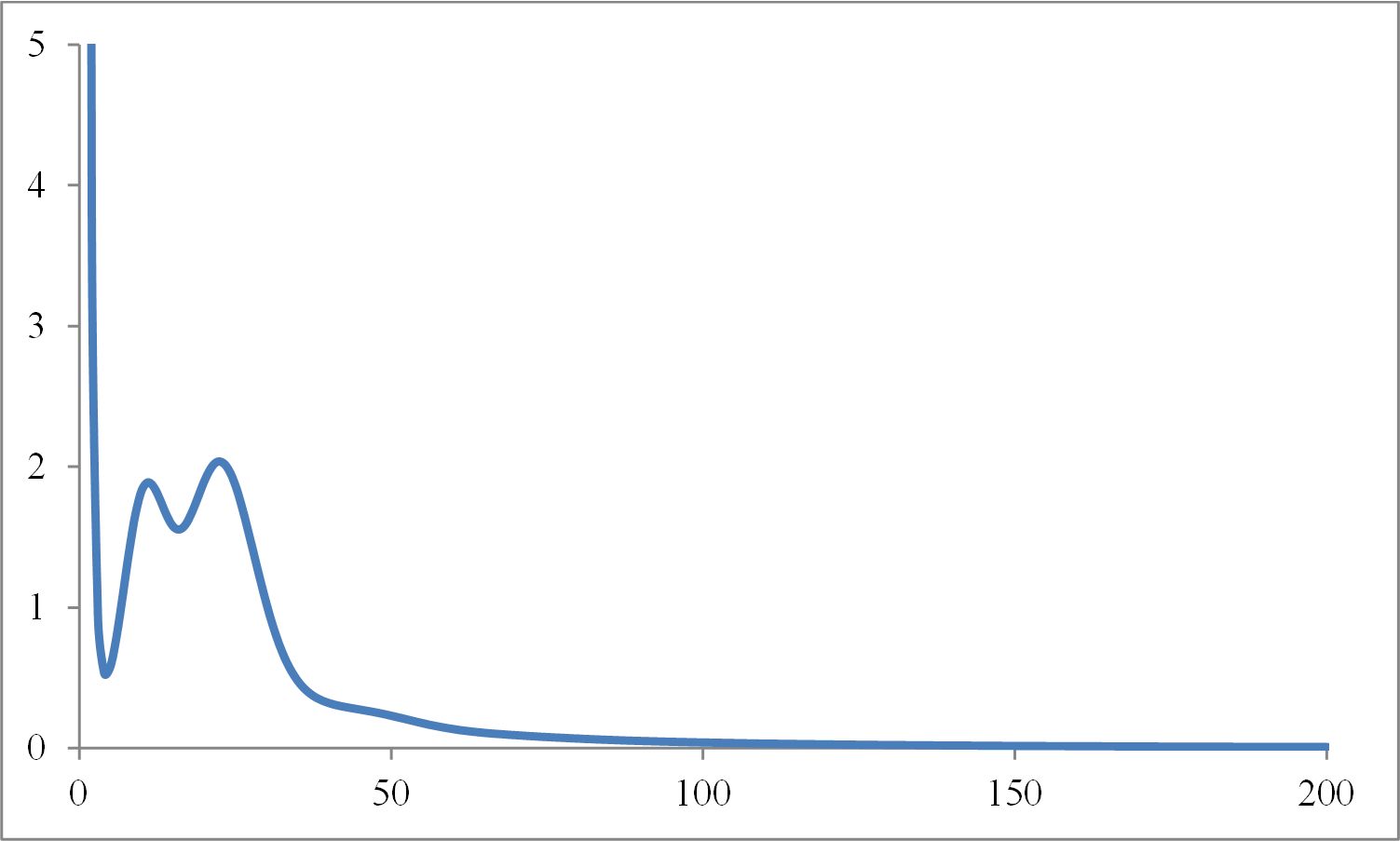


Frequency

*k*-mer occurrency

Supplementary Figure S6: The distribution of 17-mer to estimate the genome size of *D. jenkinsiana*.

##
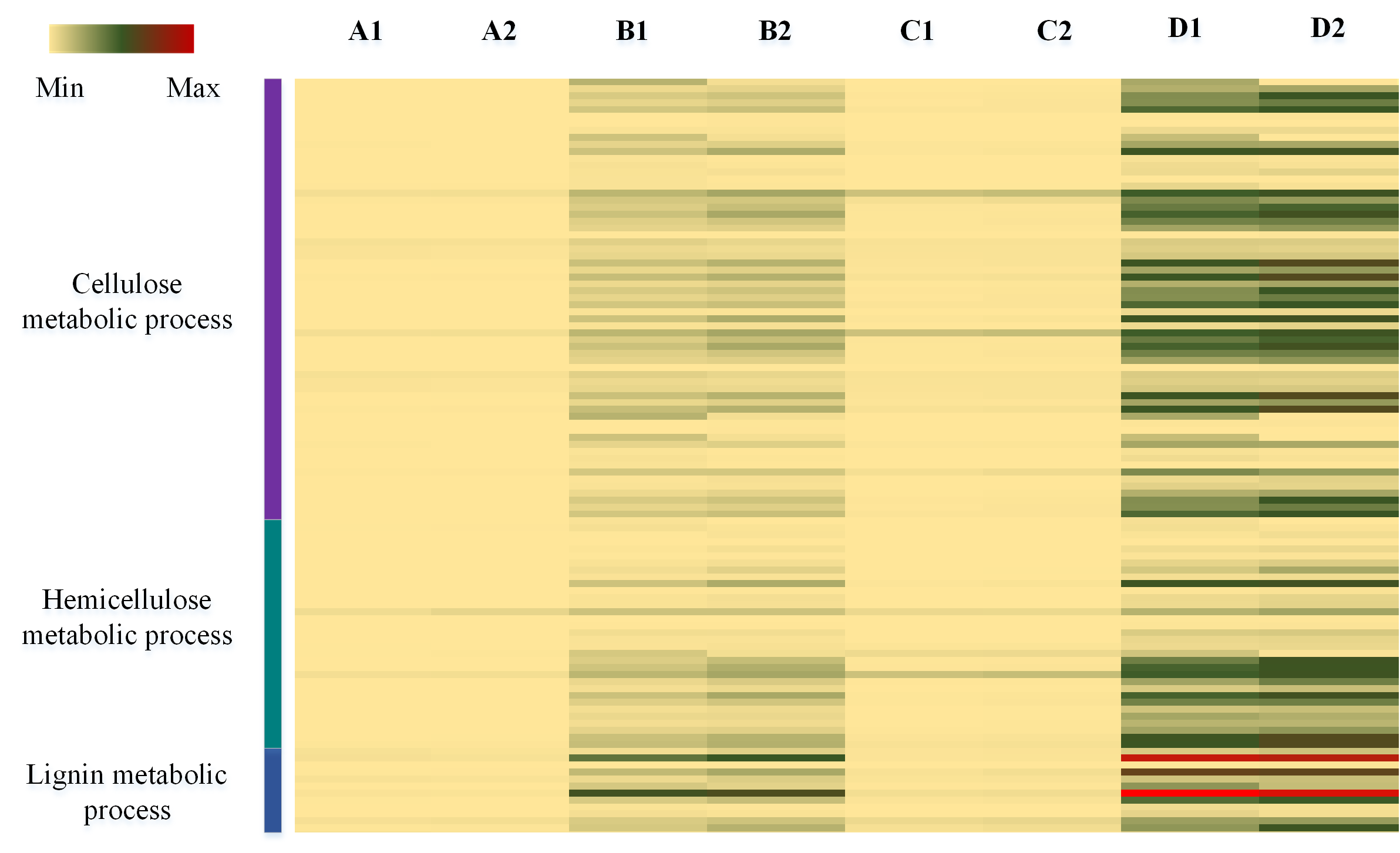


Supplementary Figure S7: Heat map of the differentially expressed genes involved in the metabolic processes of cellulose, hemicellulose, and lignin.

A: CI sample, B: CD sample, C: HI sample, and D: HD sample. 1: replicate 1; and 2: replicate 2**.**

Supplementary Table S1: the statistics for transcriptomic libraries in rattan

| Experiment Code | Library SRA Code | Strand Specificity Orientation | Insert Size Mean (bp) | Insert Size STDDEV (bp) | Raw Read Length (bp) | Raw Read Pairs Number | Clean Read Pairs Number | Effective Depth^***^ | Mapping ratio |
| --- | --- | --- | --- | --- | --- | --- | --- | --- | --- |
| CI_rep1^*^ | SRR3089417 | RF^**^ | 199 | 40 | 125 | 19,142,416 | 19,039,715 | 45.31 | 81.11% |
| CI_rep2 | SRR3089429 | RF | 191 | 39 | 125 | 21,319,410 | 21,034,006 | 48.05 | 82.12% |
| CD_rep1 | SRR3089432 | RF | 191 | 39 | 125 | 22,427,229 | 21,794,295 | 49.78 | 77.08% |
| CD_rep2 | SRR3089433 | RF | 196 | 42 | 125 | 21,127,199 | 20,849,507 | 48.87 | 79.52% |
| HI_rep1 | SRR3089434 | RF | 195 | 40 | 125 | 21,353,929 | 21,101,616 | 49.21 | 81.44% |
| HI_rep2 | SRR3089435 | RF | 185 | 38 | 125 | 21,485,412 | 21,160,188 | 46.82 | 82.34% |
| HD_rep1 | SRR3089436 | RF | 187 | 42 | 125 | 21,800,119 | 21,030,666 | 47.03 | 78.25% |
| HD_rep2 | SRR3089437 | RF | 196 | 40 | 125 | 23,678,093 | 23,365,336 | 54.77 | 80.66% |

^*^ rep1 and rep2 represented replicate 1 and 2, respectively.

^**^RF: first read of fragment pair is sequenced as anti-sense (reverse(R)), and second read is in the sense strand (forward(F)).

^***^Effective Depth = Insert Size×Clean Read Pairs Number / transcriptome size. Of them, transcriptome size was equal to the total size of Unigene (83,617,731bp).

Supplementary Table S2: Summary of the output statistics from selected tissues in *D. jenkinsiana*

| Samples | Total Clean Reads | Total Clean Nucleotides (bp) | Quality score | GC |
| --- | --- | --- | --- | --- |
| CI_rep1^*^ | 38,284,832 | 4,709,034,336 | 35.60 | 46% |
| CI_rep2 | 42,638,820 | 5,201,936,040 | 35.63 | 46% |
| CD_rep1 | 44,854,458 | 5,427,389,418 | 35.83 | 47% |
| CD_rep2 | 42,254,398 | 5,197,290,954 | 35.64 | 46% |
| HI_rep1 | 42,707,858 | 5,124,942,960 | 35.65 | 46% |
| HI_rep2 | 42,970,824 | 5,242,440,528 | 35.39 | 48% |
| HD_rep1 | 43,600,238 | 5,275,628,798 | 35.79 | 49% |
| HD_rep2 | 47,356,186 | 5,635,386,134 | 35.30 | 48% |
| Total | 344,667,614 | 41,814,049,168 | -- | -- |
| Average | 43,083,452 | 5,226,756,146 | 35.60 | 47% |

^*^rep1 and rep2 represented replicate 1 and 2, respectively.

We have produced the three biological replicated data by random sampled from the initial two biological replicates. As the third biological replicate, the dataset of CI-R3, CD-R3, HI-R3, and HD-R3 were randomly sampled into 50 M reads from the sequenced data. The method of building the third biological replicate data was based on our Perl script. The correlation coefficients between random sampling and the sequencing were all greater than 0.90, indicating that the random sampling procedure was consistent and accurate. Moreover, the reads randomness showed that the stable distribution was found in all random sampling data as well. To evaluate the difference based on two biological replicates and three biological replicates for our data, we have performed the analysis of DEGs. The summary of DEGs was provided in Supplementary Table S3. Through comparative analysis between the two datasets (see Supplementary Table S4), the predicated accuracy based on the three biological replicates is up to 98% compared with that of two biological replicates. In total, the result indicated these were high similarity based on the two biological replicates and the three biological replicates in our sequencing data.

Supplementary Table S3: The summary of the DEGs in two datasets

|  | Up-regulated | Down-regulated |
| --- | --- | --- |
| The two biological replicates by the sequencing | | |
| CI *vs.* CD | 29 | 799 |
| HI *vs.* HD | 772 | 2210 |
| The three biological replicates by the random sampling | | |
| CI *vs*. CD | 32 | 763 |
| HI *vs.* HD | 770 | 2253 |

Supplementary Table S4: The comparative analysis of the two biological replicates and the three ones

| TP* | FP* | TN* | FN* | Sensitivity | Specificity | **Accuracy** |
| --- | --- | --- | --- | --- | --- | --- |
| 57,757 | 42 | 3709 | 61 | 99.89% | 59.22% | **98.90%** |

*TP represents True Positive, an TP event indicates one gene was not DEG both in three biological replicates and two three biological replicates; FP represents False Positive, an FP event indicated one gene was DEG in three biological replicates, but was not DEG in two biological replicates; TN represents True Negative, an TN event indicated one gene was DEG both in three biological replicates and two three biological replicates; FN represents False Negative, an FN event indicated one gene was not DEG in three biological replicates, but was DEG in two biological replicates.

Supplementary Table S5: Summary of *de novo* sequence assembly by trinity software

|  | All transcript contigs | Longest isoform per gene |
| --- | --- | --- |
| Contig N10 | 4,440 | 3,895 |
| Contig N20 | 3,314 | 2,680 |
| Contig N30 | 2,617 | 1,936 |
| Contig N40 | 2,079 | 1,386 |
| Contig N50 | 1,640 | 976 |
| Median contig length | 492 | 373 |
| Average contig | 934 | 667 |
| Total assembled bases | 377,994,723 | 167,705,278 |

Supplementary Table S6: Summary of trinity genes and unigenes

|  | | Trinity genes | Unigenes |
| --- | --- | --- | --- |
| Number | | 251,468 | 61,569 |
| Size | | 167,705,278 | 83,617,731 |
| Average length | | 667 | 1,358 |
| Longest length | | 15,633 | 15,633 |
| Shortest length | | 224 | 224 |
| GC% | | 41.11 | 41.75 |
| >=1 kb | number | 39,568 | 33,426 |
|  | size | 82,733,595 | 66,917,433 |
| >=2 kb | number | 15,053 | 11,662 |
|  | size | 48,560,633 | 35,987,992 |
| >=3 kb | number | 6,579 | 4,442 |
|  | size | 28,014,349 | 18,603,027 |

Supplementary Table S7: The all sequence of unigenes (both protein and nucleotide sequences)

(Please see the supplementary fasta file)

Supplementary Table S8: The completeness of unigenes by BUSCO

| C | | F | M | Total |
| --- | --- | --- | --- | --- |
| S | D |
| 1,048 | 53 | 176 | 163 | 1,440 |
| 72.8% | 3.7% | 12.2% | 11.3% | 100% |

C: Complete BUSCOs

S: Complete and single-copy BUSCOs

D: Complete and duplicated BUSCOs

F: Fragmented BUSCOs

M: Missing BUSCOs

Supplementary Table S9: List of 10 genes isolated and 2 genes failed to isolate by RT-PCR

| **Code** | **Gene name** | **GenBank accession number** | **Forward primer sequence(5′-3′)** | **Reverse primer sequence(5′-3′)** | **Amplification length(bp)** | **Open reading frame (bp)** | **Identity**  **(%)** | **Annotation** |
| --- | --- | --- | --- | --- | --- | --- | --- | --- |
| 1 | DjSIPL2 | KU363032 | GATGGGCGCGATTAAGGCTG | ATCTCATGCTTTTTTAGCCTTGGC | 727 | 723 | 99.45% | Aquaporin SIP1-2-like |
| 2 | DjPAL1 | KU363033 | GATGCTGGTCCGGATCAACAC | CAACGGCTTCTCTCAGCACAAG | 1687 | 1674 | 99.23% | Phenylalanine ammonialyase |
| 3 | DjNAC1 | KU363034 | TAGCTTCTGCACCAAAATATGGG | GTAGGAATTCCGATCTAATTTGGC | 629 | 597 | 94.91% | NAC transcription factor 29-like |
| 4 | DjCAD1 | KU363035 | GGACGAAGAGTGATGGAGCAATC | AACTTCGAATCATTTAACCCGATC | 1090 | 1065 | 97.71% | Cinnamyl alcohol dehydrogenase |
| 5 | DjCSE1 | KU363036 | CTAATCACGCTTCATCGGATCG | CTTCTTTCGTCGTCAAGCTCCTC | 1010 | 975 | 96.53% | Caffeoyl Shikimate Esterase (CSE) |
| 6 | DjBCH1 | KU363037 | CTCTGAGGGAGGAGGAGGAGAG | ACAACAACAAATCCTCAGCCGC | 1014 | 975 | 96.15% | Beta-carotene 3-hydroxylase |
| 7 | DjSCL6 | KU363038 | ATGCTCTCGGAGTCTGCCGCCTC | TCAGCACTTCCAAGCTGACACCG | 1884 | 1884 | 100.00% | GRAS scarecrow-like protein 6 |
| 8 | DjNIP1 | KU363039 | ATGGTGGGAATGGGCGACGAC | TCACCTTCTAAAGCTTCGTACAG | 867 | 867 | 100.00% | Aquaporin NIP6-1-like |
| 9 | Djlhcb1 | KU363040 | TCACTCCCATGGCGCTGCTGTTG | ACTTCTATCTTACACAGATTTGATC | 1028 | 1011 | 98.35% | Chlorophyll a-b binding protein of LHCII type 1 |
| 10 | Djlhcb3 | KU363041 | ACGCAGATGGCGGCGACGTTAAC | TTAAGAGCCAGGCACAAACTTGGT | 819 | 813 | 99.27% | Chlorophyll a-b binding protein of LHCII type 3 |
| 11 | DjLAC1* | -- | GAGAGAGATGGAGTTGCTGCTGTG | CCAGATCATAGCGTGCTTCCAG | -- | 1736 | -- | Lccase-15-like |
| 12 | DjCCR1* | -- | GCAATGGCCGGAACAGCAACACC | GGCTAGACATATTAAATTTGTTG | -- | 1037 | -- | Cinnamoyl-CoA reductase |

* represents the genes failed to isolate from *Daemonorops jenkinsiana*

# Supplementary Table S10: The result of Gene Ontology classification

| **GO terms** | | **Number of genes** | **Percent of genes** |
| --- | --- | --- | --- |
| biological_process | biological adhesion | 132 | 0.005013102 |
| biological_process | biological regulation | 5001 | 0.189928221 |
| biological_process | cell killing | 5 | 0.00018989 |
| biological_process | cellular component organization or biogenesis | 2996 | 0.113782234 |
| biological_process | cellular process | 15367 | 0.583608674 |
| biological_process | developmental process | 1906 | 0.072386161 |
| biological_process | establishment of localization | 3673 | 0.139493373 |
| biological_process | growth | 392 | 0.014887395 |
| biological_process | immune system process | 394 | 0.014963351 |
| biological_process | localization | 3786 | 0.143784892 |
| biological_process | locomotion | 68 | 0.002582507 |
| biological_process | metabolic process | 16568 | 0.629220311 |
| biological_process | multi-organism process | 663 | 0.025179446 |
| biological_process | multicellular organismal process | 1935 | 0.073487524 |
| biological_process | negative regulation of biological process | 777 | 0.029508944 |
| biological_process | positive regulation of biological process | 658 | 0.024989556 |
| biological_process | regulation of biological process | 4675 | 0.177547378 |
| biological_process | reproduction | 1029 | 0.039079412 |
| biological_process | reproductive process | 950 | 0.036079146 |
| biological_process | response to stimulus | 3824 | 0.145228058 |
| biological_process | rhythmic process | 59 | 0.002240705 |
| biological_process | signaling | 1505 | 0.057156963 |
| biological_process | single-organism process | 7940 | 0.301545707 |
| cellular_component | cell | 11955 | 0.454027572 |
| cellular_component | cell junction | 375 | 0.014241768 |
| cellular_component | cell part | 11955 | 0.454027572 |
| cellular_component | extracellular matrix | 23 | 0.000873495 |
| cellular_component | extracellular matrix part | 9 | 0.000341802 |
| cellular_component | extracellular region | 557 | 0.021153773 |
| cellular_component | extracellular region part | 159 | 0.00603851 |
| cellular_component | macromolecular complex | 3206 | 0.121757624 |
| cellular_component | membrane | 5852 | 0.222247541 |
| cellular_component | membrane part | 3675 | 0.139569329 |
| cellular_component | membrane-enclosed lumen | 809 | 0.030724241 |
| cellular_component | nucleoid | 37 | 0.001405188 |
| cellular_component | organelle | 9618 | 0.365272872 |
| cellular_component | organelle part | 3631 | 0.137898295 |
| cellular_component | symplast | 330 | 0.012532756 |
| cellular_component | synapse | 41 | 0.0015571 |
| cellular_component | synapse part | 30 | 0.001139341 |
| cellular_component | virion | 101 | 0.003835783 |
| cellular_component | virion part | 101 | 0.003835783 |
| molecular_function | antioxidant | 177 | 0.006722115 |
| molecular_function | binding | 16703 | 0.634347347 |
| molecular_function | catalytic | 12732 | 0.483536516 |
| molecular_function | channel regulator | 3 | 0.000113934 |
| molecular_function | electron carrier | 160 | 0.006076488 |
| molecular_function | enzyme regulator | 365 | 0.013861988 |
| molecular_function | metallochaperone | 5 | 0.00018989 |
| molecular_function | molecular transducer | 397 | 0.015077285 |
| molecular_function | nucleic acid binding transcription factor | 687 | 0.026090919 |
| molecular_function | protein binding transcription factor | 118 | 0.00448141 |
| molecular_function | receptor | 250 | 0.009494512 |
| molecular_function | receptor regulator | 0 | 0 |
| molecular_function | structural molecule | 850 | 0.032281341 |
| molecular_function | translation regulator | 4 | 0.000151912 |
| molecular_function | transporter | 1554 | 0.059017888 |

# Supplementary Table S11: The result of COG annotation of putative proteins

| **Code** | **Functional-Categories** | **Gene Number** | **%** |
| --- | --- | --- | --- |
| A | RNA processing and modification | 233 | 1.09% |
| B | Chromatin structure and dynamics | 187 | 0.87% |
| C | Energy production and conversion | 1044 | 4.86% |
| D | Cell cycle control, cell division, chromosome partitioning | 524 | 2.44% |
| E | Amino acid transport and metabolism | 1181 | 5.50% |
| F | Nucleotide transport and metabolism | 242 | 1.13% |
| G | Carbohydrate transport and metabolism | 1117 | 5.20% |
| H | Coenzyme transport and metabolism | 526 | 2.45% |
| I | Lipid transport and metabolism | 639 | 2.98% |
| J | Translation, ribosomal structure and biogenesis | 1336 | 6.22% |
| K | Transcription | 1826 | 8.50% |
| L | Replication, recombination and repair | 1800 | 8.38% |
| M | Cell wall/membrane/envelope biogenesis | 496 | 2.31% |
| N | Cell motility | 64 | 0.30% |
| O | Posttranslational modification, protein turnover, chaperones | 1705 | 7.94% |
| P | Inorganic ion transport and metabolism | 801 | 3.73% |
| Q | Secondary metabolites biosynthesis, transport and catabolism | 644 | 3.00% |
| R | General function prediction only | 3948 | 18.39% |
| S | Function unknown | 703 | 3.27% |
| T | Signal transduction mechanisms | 1531 | 7.13% |
| U | Intracellular trafficking, secretion, and vesicular transport | 367 | 1.71% |
| V | Defense mechanisms | 222 | 1.03% |
| W | Extracellular structures | 0 | 0.00% |
| Y | Nuclear structure | 7 | 0.03% |
| Z | Cytoskeleton | 331 | 1.54% |

# Supplementary Table S12: The KEGG pathway enrichment analysis

| **KEGG ID** | **Pathway Name** | | | **Study. Term** | **Study. Total** | **Pop. Term** | **Pop. Total** | **p value** | **adjusted p value** |
| --- | --- | --- | --- | --- | --- | --- | --- | --- | --- |
| ko00940 | Metabolism | Biosynthesis of other secondary metabolites | Phenylpropanoid biosynthesis | 73 | 1874 | 167 | 9150 | 6.46E-12 | 1.85E-09 |
| ko00941 | Metabolism | Biosynthesis of other secondary metabolites | Flavonoid biosynthesis | 27 | 1874 | 43 | 9150 | 1.81E-09 | 2.60E-07 |
| ko00195 | Metabolism | Energy metabolism | Photosynthesis | 35 | 1874 | 66 | 9150 | 4.56E-09 | 4.36E-07 |
| ko04015 | Environmental Information Processing | Signal transduction | Rap1 signaling pathway | 34 | 1874 | 64 | 9150 | 7.13E-09 | 5.12E-07 |
| ko04540 | Cellular Processes | Cellular community | Gap junction | 26 | 1874 | 47 | 9150 | 1.45E-07 | 8.31E-06 |
| ko00945 | Metabolism | Biosynthesis of other secondary metabolites | Stilbenoid, diarylheptanoid and gingerol biosynthesis | 23 | 1874 | 40 | 9150 | 2.96E-07 | 1.42E-05 |
| ko00196 | Metabolism | Energy metabolism | Photosynthesis - antenna proteins | 18 | 1874 | 28 | 9150 | 5.86E-07 | 2.40E-05 |
| ko04145 | Cellular Processes | Transport and catabolism | Phagosome | 57 | 1874 | 155 | 9150 | 1.73E-06 | 6.21E-05 |
| ko00360 | Metabolism | Amino acid metabolism | Phenylalanine metabolism | 27 | 1874 | 59 | 9150 | 1.05E-05 | 2.88E-04 |
| ko00625 | Metabolism | Xenobiotic biodegradation and metabolism | Chloroalkane and chloroalkene degradation | 20 | 1874 | 38 | 9150 | 1.11E-05 | 2.88E-04 |
| ko00903 | Metabolism | Metabolism of terpenoids and polyketides | Limonene and pinene degradation | 23 | 1874 | 47 | 9150 | 1.20E-05 | 2.88E-04 |
| ko04713 | Organismal Systems | Environmental adaptation | Circadian entrainment | 20 | 1874 | 39 | 9150 | 1.84E-05 | 4.07E-04 |
| ko00500 | Metabolism | Carbohydrate metabolism | Starch and sucrose metabolism | 84 | 1874 | 274 | 9150 | 3.49E-05 | 6.27E-04 |
| ko04014 | Environmental Information Processing | Signal transduction | Ras signaling pathway | 34 | 1874 | 86 | 9150 | 3.81E-05 | 6.44E-04 |
| ko04740 | Organismal Systems | Sensory system | Olfactory transduction | 14 | 1874 | 24 | 9150 | 5.30E-05 | 7.24E-04 |
| ko04925 | Organismal Systems | Endocrine system | Aldosterone synthesis and secretion | 14 | 1874 | 24 | 9150 | 5.30E-05 | 7.24E-04 |
| ko04971 | Organismal Systems | Digestive system | Gastric acid secretion | 14 | 1874 | 24 | 9150 | 5.30E-05 | 7.24E-04 |
| ko00380 | Metabolism | Amino acid metabolism | Tryptophan metabolism | 28 | 1874 | 67 | 9150 | 5.67E-05 | 7.39E-04 |
| ko00520 | Metabolism | Carbohydrate metabolism | Amino sugar and nucleotide sugar metabolism | 70 | 1874 | 225 | 9150 | 9.30E-05 | 1.16E-03 |
| ko04744 | Organismal Systems | Sensory system | Phototransduction | 14 | 1874 | 25 | 9150 | 9.78E-05 | 1.17E-03 |
| ko00040 | Metabolism | Carbohydrate metabolism | Pentose and glucuronate interconversions | 36 | 1874 | 97 | 9150 | 1.09E-04 | 1.25E-03 |
| ko00630 | Metabolism | Carbohydrate metabolism | Glyoxylate and dicarboxylate metabolism | 53 | 1874 | 161 | 9150 | 1.33E-04 | 1.46E-03 |
| ko04915 | Organismal Systems | Endocrine system | Estrogen signaling pathway | 43 | 1874 | 124 | 9150 | 1.52E-04 | 1.57E-03 |
| ko04970 | Organismal Systems | Digestive system | Salivary secretion | 13 | 1874 | 23 | 9150 | 1.54E-04 | 1.57E-03 |
| ko00710 | Metabolism | Energy metabolism | Carbon fixation in photosynthetic organisms | 46 | 1874 | 136 | 9150 | 1.78E-04 | 1.76E-03 |
| ko00260 | Metabolism | Amino acid metabolism | Glycine, serine and threonine metabolism | 36 | 1874 | 104 | 9150 | 5.28E-04 | 5.05E-03 |
| ko04810 | Cellular Processes | Cell motility | Regulation of actin cytoskeleton | 47 | 1874 | 148 | 9150 | 7.65E-04 | 7.08E-03 |
| ko01200 | Metabolism | Overview | Carbon metabolism | 138 | 1874 | 529 | 9150 | 8.01E-04 | 7.18E-03 |
| ko00561 | Metabolism | Lipid metabolism | Glycerolipid metabolism | 38 | 1874 | 119 | 9150 | 2.10E-03 | 1.79E-02 |
| ko04912 | Organismal Systems | Endocrine system | GnRH signaling pathway | 22 | 1874 | 59 | 9150 | 2.12E-03 | 1.79E-02 |
| ko00270 | Metabolism | Amino acid metabolism | Cysteine and methionine metabolism | 43 | 1874 | 139 | 9150 | 2.22E-03 | 1.82E-02 |
| ko00982 | Metabolism | Xenobiotics biodegradation and metabolism | Drug metabolism - cytochrome P450 | 26 | 1874 | 74 | 9150 | 2.36E-03 | 1.88E-02 |
| ko00250 | Metabolism | Amino acid metabolism | Alanine, aspartate and glutamate metabolism | 32 | 1874 | 97 | 9150 | 2.57E-03 | 1.99E-02 |
| ko05133 | Human Diseases | Infectious diseases | Pertussis | 44 | 1874 | 147 | 9150 | 3.98E-03 | 3.01E-02 |
| ko00340 | Metabolism | Amino acid metabolism | Histidine metabolism | 17 | 1874 | 44 | 9150 | 4.29E-03 | 3.16E-02 |
| ko00410 | Metabolism | Metabolism of other amino acids | beta-Alanine metabolism | 28 | 1874 | 85 | 9150 | 4.72E-03 | 3.31E-02 |
| ko04924 | Organismal Systems | Endocrine system | Renin secretion | 17 | 1874 | 45 | 9150 | 5.61E-03 | 3.83E-02 |
| ko00330 | Metabolism | Amino acid metabolism | Arginine and proline metabolism | 33 | 1874 | 106 | 9150 | 6.14E-03 | 4.00E-02 |
| ko00053 | Metabolism | Carbohydrate metabolism | Ascorbate and aldarate metabolism | 29 | 1874 | 91 | 9150 | 6.88E-03 | 4.39E-02 |
| ko04972 | Organismal Systems | Digestive system | Pancreatic secretion | 20 | 1874 | 57 | 9150 | 7.30E-03 | 4.45E-02 |
| ko01220 | Metabolism | Overview | Degradation of aromatic compounds | 8 | 1874 | 16 | 9150 | 8.08E-03 | 4.83E-02 |
| ko00980 | Metabolism | Xenobiotics biodegradation and metabolism | Metabolism of xenobiotics by cytochrome P450 | 23 | 1874 | 69 | 9150 | 8.46E-03 | 4.86E-02 |

# Supplementary Table S13: The result of species distribution via the comparative analysis of homologous genes

| Species | % | Species | % |
| --- | --- | --- | --- |
| *Elaeis guineensis* | 11.00% | *Megachile rotundata* | 0.39% |
| *Phoenix dactylifera* | 10.20% | *Harpegnathos saltator* | 0.37% |
| *Musa acuminata subsp. malaccensis* | 7.99% | *Triticum urartu* | 0.36% |
| *Nelumbo nucifera* | 4.23% | *Cerapachys biroi* | 0.36% |
| *Vitis vinifera* | 4.21% | *Camponotus floridanus* | 0.35% |
| *Zea mays* | 2.50% | *Orussus abietinus* | 0.35% |
| *Oryza sativa Japonica Group* | 2.24% | *Acromyrmex echinatior* | 0.34% |
| *Gossypium raimondii* | 1.78% | *Camelina sativa* | 0.34% |
| *Theobroma cacao* | 1.72% | *Tarenaya hassleriana* | 0.33% |
| *Citrus sinensis* | 1.69% | *Apis mellifera* | 0.32% |
| *Setaria italica* | 1.46% | *Solenopsis invicta* | 0.32% |
| *Jatropha curcas* | 1.35% | *Bombus terrestris* | 0.31% |
| *Acyrthosiphon pisum* | 1.33% | *Bombus impatiens* | 0.31% |
| *Populus euphratica* | 1.31% | *Nasonia vitripennis* | 0.30% |
| *Populus trichocarpa* | 1.23% | *Zostera marina* | 0.30% |
| *Brachypodium distachyon* | 1.18% | *Fopius arisanus* | 0.30% |
| *Oryza brachyantha* | 1.09% | *Apis dorsata* | 0.27% |
| *Malus domestica* | 1.06% | *Wasmannia auropunctata* | 0.27% |
| *Eucalyptus grandis* | 1.05% | *Pogonomyrmex barbatus* | 0.27% |
| *Prunus mume* | 1.04% | *Linepithema humile* | 0.27% |
| *Amborella trichopoda* | 1.03% | *Monomorium pharaonis* | 0.27% |
| *Pyrus x bretschneideri* | 1.03% | *Microplitis demolitor* | 0.26% |
| *Citrus clementina* | 1.00% | *Vollenhovia emeryi* | 0.26% |
| *Sorghum bicolor* | 0.98% | *Brassica napus* | 0.25% |
| *Prunus persica* | 0.97% | *Arabidopsis thaliana* | 0.25% |
| *Glycine max* | 0.97% | *Apis florea* | 0.24% |
| *Oryza sativa Indica Group* | 0.93% | *Triticum aestivum* | 0.22% |
| *Sesamum indicum* | 0.89% | *Pediculus humanus corporis* | 0.20% |
| *Ricinus communis* | 0.83% | *Dendroctonus ponderosae* | 0.19% |
| *Nicotiana sylvestris* | 0.72% | *Ceratosolen solmsi marchali* | 0.19% |
| *Hordeum vulgare subsp. vulgare* | 0.70% | *Brassica rapa* | 0.18% |
| *Nicotiana tomentosiformis* | 0.69% | *Diaphorina citri* | 0.18% |
| *Morus notabilis* | 0.67% | *Atta cephalotes* | 0.16% |
| *Fragaria vesca subsp. vesca* | 0.60% | *Bombyx mori* | 0.15% |
| *Cucumis sativus* | 0.57% | *Aedes aegypti* | 0.15% |
| *Gossypium arboreum* | 0.56% | *Papilio xuthus* | 0.15% |
| *Coffea canephora* | 0.56% | *Spinacia oleracea* | 0.14% |
| *Medicago truncatula* | 0.51% | *Eutrema salsugineum* | 0.14% |
| *Cucumis melo* | 0.50% | *Amyelois transitella* | 0.13% |
| *Aegilops tauschii* | 0.49% | *Erythranthe guttata* | 0.13% |
| *Solanum lycopersicum* | 0.47% | *Plutella xylostella* | 0.13% |
| *Solanum tuberosum* | 0.45% | *Papilio polytes* | 0.12% |
| *Tribolium castaneum* | 0.45% | *Lotus japonicus* | 0.12% |
| *Beta vulgaris subsp. vulgaris* | 0.45% | *Capsella rubella* | 0.11% |
| *Erythranthe guttatus* | 0.44% | *ubiquinone* | 0.11% |
| *Phaseolus vulgaris* | 0.42% | *Lasius niger* | 0.11% |
| *Cicer arietinum* | 0.41% | *Anopheles gambiae str. PEST* | 0.11% |
| *Glycine soja* | 0.41% | *Arabidopsis lyrata subsp. lyrata* | 0.10% |
| *Athalia rosae* | 0.41% | *Culex quinquefasciatus* | 0.10% |
| *Zootermopsis nevadensis* | 0.41% | *Danaus plexippus* | 0.10% |

# Supplementary Table S14: The list of the up-regulated genes in CI *vs.* CD

| **No.** | **id** | **logFC** | **logCPM** | **PValue** | **FDR** |
| --- | --- | --- | --- | --- | --- |
| 1 | TR93100_c0_g3 | -9.720105 | 5.8412456 | 7.94E-25 | 2.32E-20 |
| 2 | TR88370_c0_g2 | -7.888289 | 12.408283 | 5.11E-22 | 2.99E-18 |
| 3 | TR91291_c0_g1 | -7.128492 | 5.2860272 | 7.55E-13 | 3.87E-10 |
| 4 | TR68949_c0_g1 | -5.489147 | 7.4317299 | 6.69E-12 | 2.62E-09 |
| 5 | TR86556_c0_g1 | -5.716203 | 4.9595069 | 1.03E-11 | 3.88E-09 |
| 6 | TR68648_c0_g1 | -5.087336 | 4.4985252 | 3.32E-10 | 9.25E-08 |
| 7 | TR82203_c0_g1 | -3.695581 | 8.7681422 | 3.61E-10 | 9.90E-08 |
| 8 | TR80662_c0_g1 | -3.637849 | 6.8367011 | 1.62E-09 | 3.85E-07 |
| 9 | TR88370_c0_g1 | -8.668721 | 3.0350576 | 2.36E-09 | 5.36E-07 |
| 10 | TR91615_c1_g1 | -4.002941 | 5.1957773 | 3.87E-09 | 8.38E-07 |
| 11 | TR66868_c0_g1 | -8.704877 | 3.0705153 | 6.21E-09 | 1.27E-06 |
| 12 | TR93610_c1_g1 | -3.522224 | 6.3850639 | 1.31E-08 | 2.37E-06 |
| 13 | TR47758_c0_g1 | -5.816895 | 3.9902389 | 4.69E-08 | 7.34E-06 |
| 14 | TR50186_c0_g1 | -4.794784 | 4.4464837 | 9.01E-08 | 1.30E-05 |
| 15 | TR84716_c0_g1 | -5.031347 | 3.5958591 | 1.14E-07 | 1.58E-05 |
| 16 | TR83273_c0_g1 | -6.883538 | 3.7962134 | 2.39E-07 | 2.97E-05 |
| 17 | TR72029_c0_g1 | -7.111578 | 2.8730371 | 3.18E-07 | 3.80E-05 |
| 18 | TR99395_c8_g1 | -5.65387 | 3.4541225 | 3.24E-07 | 3.85E-05 |
| 19 | TR99382_c3_g3 | -7.772414 | 2.1613817 | 5.70E-07 | 6.29E-05 |
| 20 | TR89643_c0_g2 | -5.380785 | 3.724511 | 8.65E-07 | 9.01E-05 |
| 21 | TR90901_c1_g2 | -3.740333 | 4.1566341 | 8.68E-07 | 9.01E-05 |
| 22 | TR89133_c1_g1 | -3.695039 | 4.7367601 | 9.13E-07 | 9.40E-05 |
| 23 | TR89643_c0_g1 | -3.667852 | 6.7320441 | 1.05E-06 | 0.0001068 |
| 24 | TR88584_c0_g1 | -3.436953 | 5.3726517 | 1.53E-06 | 0.0001485 |
| 25 | TR93610_c0_g1 | -3.777692 | 3.7637838 | 3.62E-06 | 0.0003085 |
| 26 | TR89657_c2_g3 | -3.229098 | 5.574198 | 7.69E-06 | 0.0005997 |
| 27 | TR80618_c2_g1 | -4.24275 | 5.8001051 | 8.67E-06 | 0.0006634 |
| 28 | TR99164_c3_g11 | -5.003336 | 3.5734883 | 9.10E-06 | 0.0006899 |
| 29 | TR82752_c0_g2 | -7.116207 | 2.3568395 | 1.02E-05 | 0.0007517 |

# Supplementary Table S15: The list of the down-regulated genes in CI *vs.* CD

(Please see the supplementary Excel file)

# Supplementary Table S16: The list of the up-regulated genes in HI *vs.* HD

(Please see the supplementary Excel file)

# Supplementary Table S17: The list of the down-regulated genes in HI *vs.* HD

(Please see the supplementary Excel file)

# Supplementary Table S18: 37 primer pairs used for qRT-PCR expression analysis for validating the expressed value

| ID | Assembly name | Primer | Tm (ºC) | PCR efficiency values | Correlation coefficients (R^2^) | Product size (bp) |
| --- | --- | --- | --- | --- | --- | --- |
| Gene1 | TR93130_c0_g5 | F: 5′-ATCCCACTCCTTCTCCATCAC-3′ | 60 | 0.95 | 0.999 | 88 |
|  |  | R: 5′-CTCCCGAGGTTCTCCTTTATC-3′ |  |  |  |  |
| Gene2 | TR80430_c1_g1 | F: 5′-TCATCCCAGGTAGGAGTTCAGT-3′ | 62 | 0.97 | 0.997 | 108 |
|  |  | R: 5′-GACCATGACCAGGGCCATCAC-3′ |  |  |  |  |
| Gene3 | TR94607_c4_g1 | F: 5′-GGTACGGCTACGGTGGGAACT-3′ | 64 | 0.96 | 0.998 | 118 |
|  |  | R: 5′-AGGCAGACGGCTGGGATTGTG-3′ |  |  |  |  |
| Gene4 | TR97389_c0_g3 | F: 5′-GAGCTTTGGTTCGGCTTCTAC-3′ | 62 | 1.02 | 0.994 | 112 |
|  |  | R: 5′-TCGGTAACTCAGTTTCATCCC-3′ |  |  |  |  |
| Gene5 | TR96617_c0_g1 | F: 5′-AAGTGGTGGGCATGATATGGA-3′ | 60 | 1.04 | 0.990 | 96 |
|  |  | R: 5′-CCAGCCTTCTTGTGGTGATTG-3′ |  |  |  |  |
| Gene6 | TR99522_c3_g1 | F: 5′-TTTGGTATGGATTCGGAGGTG-3′ | 58 | 1.04 | 0.991 | 96 |
|  |  | R: 5′-CAAGAAGAGGGAGGGATGTGA-3′ |  |  |  |  |
| Gene7 | TR85994_c0_g1 | F: 5′-CCCTACCGCATGGTGATTGTG-3′ | 58 | 0.99 | 0.995 | 80 |
|  |  | R: 5′-TCGTGGACTGGGTTGAGGATT-3′ |  |  |  |  |
| Gene8 | TR95918_c2_g1 | F: 5′-CCCACCTGTTTGCTGTCATTC-3′ | 62 | 0.96 | 0.995 | 94 |
|  |  | R: 5′-TCCTCATCGTCTGTAGCCTTG-3′ |  |  |  |  |
| Gene9 | TR99522_c4_g13 | F: 5′-TGATGACCAGGATGAAGGAGG-3′ | 62 | 0.98 | 0.991 | 88 |
|  |  | R: 5′-TGGGTTCCCAGATTCACCATT-3′ |  |  |  |  |
| Gene10 | TR95918_c3_g1 | F: 5′-AAGATGAGTTCCCACTAAAGAGCC-3′ | 62 | 0.96 | 0.992 | 79 |
|  |  | R: 5′-CGGTCACCATTCTCCGAGTTA-3′ |  |  |  |  |
| Gene11 | TR97320_c8_g2 | F: 5′-TACCCTCTACAAAGATCCCTAATCC-3′ | 62 | 1.02 | 1.000 | 105 |
|  |  | R: 5′-CTCCATCATCCTCGTCCAACT-3′ |  |  |  |  |
| Gene12 | TR75613_c0_g1 | F: 5′-CTGGCATCTCCAACTCACTAT-3′ | 60 | 1.05 | 1.000 | 94 |
|  |  | R: 5′-GCCTCTGAACAATCTTCACCC-3′ |  |  |  |  |
| Gene13 | TR83900_c0_g1 | F: 5′-ACAAGGATACAGAAGATGTCGCTAG-3′ | 60 | 1.01 | 0.999 | 88 |
|  |  | R: 5′-CGTTGAAACCGAAGAAGGAATAG-3′ |  |  |  |  |
| Gene14 | TR98396_c1_g3 | F: 5′-CCATTCACATCCCTCCCTCTT-3′ | 62 | 0.96 | 0.998 | 118 |
|  |  | R: 5′-ACAGCCCAAGGAACCAGACAC-3′ |  |  |  |  |
| Gene15 | TR32793_c0_g1 | F: 5′-ATTGGCATCAGTTTTCTCCCT-3′ | 60 | 1.05 | 1.000 | 93 |
|  |  | R: 5′-TCGATAGAAATGCAACCTTCAG-3′ |  |  |  |  |
| Gene16 | TR97320_c3_g1 | F: 5′-CAACACTGGCATCTCATCTCC-3′ | 58 | 0.97 | 0.997 | 100 |
|  |  | R: 5′-GATTCACCATTTGCCTCACTT-3′ |  |  |  |  |
| Gene17 | TR99522_c3_g4 | F: 5′-TGGGTTATGCAAGATGGAACA-3′ | 60 | 0.96 | 0.998 | 92 |
|  |  | R: 5′-CAAGCACCACCATATCCAAGA-3′ |  |  |  |  |
| Gene18 | TR95918_c1_g1 | F: 5′-GTGAGAAGCGGCCTGGATTT-3′ | 60 | 1.04 | 0.997 | 95 |
|  |  | R: 5′-CATGAAGGGTGCGTTTGTGAG-3′ |  |  |  |  |
| Gene19 | TR59287_c0_g1 | F: 5′-AAGATGCACTGCCGTGGATG-3′ | 60 | 1.04 | 0.990 | 84 |
|  |  | R: 5′-AGAAAGGTTGATGGGTGCTGA-3′ |  |  |  |  |
| Gene20 | TR40219_c0_g1 | F: 5′-GAATAATCCCCGAGACCACCC-3′ | 60 | 1.05 | 0.994 | 96 |
|  |  | R: 5′-TAGACTAGCCGAGGAAGAACA-3′ |  |  |  |  |
| Gene21 | TR95250_c2_g1 | F: 5′-TGTTTCTTGGTGGATCGGACAG-3′ | 60 | 0.99 | 0.995 | 105 |
|  |  | R: 5′-CTCGGATTTCATCTTGGACTTTC-3′ |  |  |  |  |
| Gene22 | TR90803_c0_g2 | F: 5′-CCCTCAGCCTCCCTTACTTAT-3′ | 62 | 0.96 | 0.995 | 73 |
|  |  | R: 5′-TTCATCACCCTTGTATCCTCC-3′ |  |  |  |  |
| Gene23 | TR97457_c0_g6 | F: 5′-GAAATCGGAGGTGACTGTATC-3′ | 60 | 0.98 | 0.991 | 102 |
|  |  | R: 5′-CTGTTAGCCTTTGTATGTGCC-3′ |  |  |  |  |
| Gene24 | TR77234_c0_g1 | F: 5′-CCATTCCCACTACCATTCTTCTG-3′ | 62 | 0.96 | 0.992 | 87 |
|  |  | R: 5′-TGAGCTACTGGACCATACAAACCT-3′ |  |  |  |  |
| Gene25 | TR84148_c0_g1 | F: 5′-ACAGAAGCCGGTGACGGTGGT-3′ | 63 | 1.05 | 1.000 | 70 |
|  |  | R: 5′-GTGAGCGGATTCCCTTAAACA-3′ |  |  |  |  |
| Gene26 | TR98207_c0_g4 | F: 5′-AGTCCACTGCCATAGGTCCAT-3′ | 62 | 1.05 | 1.000 | 80 |
|  |  | R: 5′-CCAGTCCAAGGCTGCCTACTT-3′ |  |  |  |  |
| Gene27 | TR86816_c1_g1 | F: 5′-GAGTTTATTGCCGTCTCCCTAG-3′ | 62 | 1.01 | 0.999 | 81 |
|  |  | R: 5′-TGCATCAGACCACCCACTACC-3′ |  |  |  |  |
| Gene28 | TR85089_c2_g1 | F: 5′-AGGGAACTAGAAAGGAAAGGG-3′ | 60 | 1.05 | 1.000 | 110 |
|  |  | R: 5′-TAGGACTGATGGGCATTGAGA-3′ |  |  |  |  |
| Gene29 | TR89397_c0_g1 | F: 5′-TCGTTCCTCCGCTTCCTCTTT-3′ | 62 | 1.04 | 0.991 | 101 |
|  |  | R: 5′-CTCTTCACTGGCGTTGCTTGG-3′ |  |  |  |  |
| Gene30 | TR63499_c0_g2 | F: 5′-TAGAGCAAAGGGATCGAAGAT-3′ | 62 | 1.03 | 0.990 | 86 |
|  |  | R: 5′-CAGTAGCGAGTAGTAGGAGACG-3′ |  |  |  |  |
| Gene31 | TR92185_c1_g1 | F: 5′-CTTCCCTCGCCTCCACTTCTT-3′ | 63 | 1.05 | 0.991 | 85 |
|  |  | R: 5′-AGGCACAGTGAGCGCCCTATA-3′ |  |  |  |  |
| Gene32 | TR44807_c0_g1 | F: 5′-GATGCAACCGCTGAAGAGGAG-3′ | 62 | 0.97 | 0.997 | 116 |
|  |  | R: 5′-GACAACGCAGAAACATAGGAG-3′ |  |  |  |  |
| Gene33 | TR92185_c1_g5 | F: 5′-AGGTGGTTCAGGTCGCCAAAG-3′ | 64 | 0.96 | 0.998 | 79 |
|  |  | R: 5′-TCGACAACGAGGCCCTCTACG-3′ |  |  |  |  |
| Gene34 | TR83459_c0_g3 | F: 5′-TGAAGCCTTGCTCATTACCCA-3′ | 60 | 1.04 | 0.992 | 119 |
|  |  | R: 5′-TGATTTCAGAGCCAACCTTTACC-3′ |  |  |  |  |
| Gene35 | TR86816_c1_g2 | F: 5′-CCTCCTCGCAAACTCCATAAATAG-3′ | 62 | 1.04 | 0.991 | 72 |
|  |  | R: 5′-AGGCCGGGTACATAGACAAAGG-3′ |  |  |  |  |
| Gene36 | TR74858_c0_g1 | F: 5′-TGTCATTTCTGGCTTTGTTGT-3′ | 58 | 1.05 | 0.994 | 91 |
|  |  | R: 5′-AAGGACCCTATTGTCTTTGCT-3′ |  |  |  |  |
| Gene37 | TR93480_c0_g1 | F: 5′-TGCATCGAATGGTCCTCTTAC-3′ | 60 | 0.99 | 0.995 | 79 |
|  |  | R: 5′-AATAAACCCGTCCGCCAAACT-3′ |  |  |  |  |
| γ-tubulin | TR97697_c2_g9 | F: 5′-ACGGGAAATTGTGAGGGACAT-3′ | 62 | 0.98 | 0.991 | 119 |
|  |  | R: 5′-AGCAGAGCTACTCTTGGCAGT-3′ |  |  |  |  |

# Supplementary Table S19: The list of the significant GO terms

| GO ID | GO terms | Significant sample | Adjusted P value |
| --- | --- | --- | --- |
| GO:0005198 | structural molecule activity | common | - |
| GO:0043228 | non-membrane-bounded organelle | common | - |
| GO:0043232 | intracellular non-membrane-bounded organelle | common | - |
| GO:0010410 | hemicellulose metabolic process | common | - |
| GO:0044036 | cell wall macromolecule metabolic process | common | - |
| GO:0015630 | microtubule cytoskeleton | Down-regulated in CI vs. CD | 1.68E-07 |
| GO:0005576 | extracellular region | Down-regulated in CI vs. CD | 1.23E-06 |
| GO:0005874 | microtubule | Down-regulated in CI vs. CD | 2.33E-06 |
| GO:0008889 | glycerophosphodiester phosphodiesterase activity | Up-regulated in HI vs. HD | 4.88E-04 |
| GO:0003824 | catalytic activity | Up-regulated in HI vs. HD | 2.26E-03 |
| GO:0016757 | transferase activity, transferring glycosyl groups | Up-regulated in HI vs. HD | 7.67E-03 |
| GO:0005856 | cytoskeleton | Down-regulated in HI vs. HD | 2.29E-18 |
| GO:0009834 | secondary cell wall biogenesis | Down-regulated in HI vs. HD | 2.56E-17 |
| GO:0044430 | cytoskeletal part | Down-regulated in HI vs. HD | 1.17E-13 |

# Supplementary Table S20: The GO terms enrichment analysis of the down-regulated genes in CI *vs.* CD

(Please see the supplementary Excel file)

# Supplementary Table S21: The GO terms enrichment analysis of the up-regulated genes in HI *vs.* HD

| **No.** | **ID** | **Pop. total** | **Pop. term** | **Study. total** | **Study. term** | **p** | **p. adjusted** | **name** |
| --- | --- | --- | --- | --- | --- | --- | --- | --- |
| 1 | GO:0008889 | 26303 | 10 | 434 | 5 | 2.81E-07 | 4.88E-04 | glycerophosphodiester phosphodiesterase activity |
| 2 | GO:0003824 | 26303 | 12731 | 434 | 259 | 1.30E-06 | 0.0022648 | catalytic activity |
| 3 | GO:0016757 | 26303 | 724 | 434 | 30 | 4.42E-06 | 0.0076699 | transferase activity, transferring glycosyl groups |

# Supplementary Table S22: The GO terms enrichment analysis of the down-regulated genes in HI *vs.* HD

| **No.** | **ID** | **Pop.**  **total** | **Pop.**  **term** | **Study.**  **total** | **Study.**  **term** | **p** | **p. adjusted** | **name** |
| --- | --- | --- | --- | --- | --- | --- | --- | --- |
| 1 | GO:0005856 | 26303 | 568 | 1370 | 91 | 6.91E-22 | 2.29E-18 | cytoskeleton |
| 2 | GO:0009834 | 26303 | 37 | 1370 | 23 | 7.72E-21 | 2.56E-17 | secondary cell wall biogenesis |
| 3 | GO:0071554 | 26303 | 540 | 1370 | 83 | 6.50E-19 | 2.15E-15 | cell wall organization or biogenesis |
| 4 | GO:0043228 | 26303 | 1758 | 1370 | 177 | 8.41E-18 | 2.78E-14 | non-membrane-bounded organelle |
| 5 | GO:0043232 | 26303 | 1758 | 1370 | 177 | 8.41E-18 | 2.78E-14 | intracellular non-membrane-bounded organelle |
| 6 | GO:0010410 | 26303 | 138 | 1370 | 38 | 9.39E-18 | 3.11E-14 | hemicellulose metabolic process |
| 7 | GO:0045491 | 26303 | 126 | 1370 | 36 | 1.83E-17 | 6.07E-14 | xylan metabolic process |
| 8 | GO:0044430 | 26303 | 446 | 1370 | 71 | 3.53E-17 | 1.17E-13 | cytoskeletal part |
| 9 | GO:0044036 | 26303 | 201 | 1370 | 45 | 5.86E-17 | 1.94E-13 | cell wall macromolecule metabolic process |
| 10 | GO:0010383 | 26303 | 165 | 1370 | 39 | 1.01E-15 | 3.34E-12 | cell wall polysaccharide metabolic process |
| 11 | GO:0005198 | 26303 | 850 | 1370 | 103 | 1.04E-15 | 3.46E-12 | structural molecule activity |
| 12 | GO:0015630 | 26303 | 331 | 1370 | 53 | 2.91E-13 | 9.63E-10 | microtubule cytoskeleton |
| 13 | GO:0042546 | 26303 | 244 | 1370 | 44 | 4.71E-13 | 1.56E-09 | cell wall biogenesis |
| 14 | GO:0071669 | 26303 | 238 | 1370 | 43 | 8.11E-13 | 2.68E-09 | plant-type cell wall organization or biogenesis |
| 15 | GO:0045492 | 26303 | 100 | 1370 | 27 | 8.46E-13 | 2.80E-09 | xylan biosynthetic process |
| 16 | GO:0009832 | 26303 | 152 | 1370 | 33 | 2.03E-12 | 6.73E-09 | plant-type cell wall biogenesis |
| 17 | GO:0005874 | 26303 | 198 | 1370 | 37 | 1.13E-11 | 3.73E-08 | microtubule |
| 18 | GO:0005200 | 26303 | 50 | 1370 | 18 | 2.62E-11 | 8.67E-08 | structural constituent of cytoskeleton |
| 19 | GO:0070592 | 26303 | 119 | 1370 | 27 | 6.98E-11 | 2.31E-07 | cell wall polysaccharide biosynthetic process |
| 20 | GO:0010413 | 26303 | 95 | 1370 | 24 | 7.21E-11 | 2.39E-07 | glucuronoxylan metabolic process |
| 21 | GO:0070589 | 26303 | 120 | 1370 | 27 | 8.56E-11 | 2.83E-07 | cellular component macromolecule biosynthetic process |
| 22 | GO:0044038 | 26303 | 120 | 1370 | 27 | 8.56E-11 | 2.83E-07 | cell wall macromolecule biosynthetic process |
| 23 | GO:0005811 | 26303 | 33 | 1370 | 14 | 3.26E-10 | 1.08E-06 | lipid particle |
| 24 | GO:0008092 | 26303 | 397 | 1370 | 52 | 9.80E-10 | 3.25E-06 | cytoskeletal protein binding |
| 25 | GO:0009698 | 26303 | 83 | 1370 | 21 | 1.07E-09 | 3.53E-06 | phenylpropanoid metabolic process |
| 26 | GO:0030529 | 26303 | 1042 | 1370 | 101 | 1.26E-09 | 4.17E-06 | ribonucleoprotein complex |
| 27 | GO:0051258 | 26303 | 137 | 1370 | 27 | 1.99E-09 | 6.58E-06 | protein polymerization |
| 28 | GO:0005576 | 26303 | 557 | 1370 | 64 | 2.72E-09 | 9.00E-06 | extracellular region |
| 29 | GO:0071555 | 26303 | 313 | 1370 | 43 | 6.56E-09 | 2.17E-05 | cell wall organization |
| 30 | GO:0003735 | 26303 | 622 | 1370 | 68 | 6.76E-09 | 2.24E-05 | structural constituent of ribosome |
| 31 | GO:0010116 | 26303 | 8 | 1370 | 7 | 7.83E-09 | 2.59E-05 | positive regulation of abscisic acid biosynthetic process |
| 32 | GO:0045828 | 26303 | 8 | 1370 | 7 | 7.83E-09 | 2.59E-05 | positive regulation of isoprenoid metabolic process |
| 33 | GO:0032991 | 26303 | 3214 | 1370 | 237 | 1.17E-08 | 3.87E-05 | macromolecular complex |
| 34 | GO:0007017 | 26303 | 378 | 1370 | 48 | 1.18E-08 | 3.89E-05 | microtubule-based process |
| 35 | GO:0005840 | 26303 | 741 | 1370 | 76 | 1.43E-08 | 4.73E-05 | ribosome |
| 36 | GO:0010115 | 26303 | 9 | 1370 | 7 | 3.36E-08 | 1.11E-04 | regulation of abscisic acid biosynthetic process |
| 37 | GO:0046889 | 26303 | 9 | 1370 | 7 | 3.36E-08 | 1.11E-04 | positive regulation of lipid biosynthetic process |
| 38 | GO:0045229 | 26303 | 332 | 1370 | 43 | 3.79E-08 | 1.25E-04 | external encapsulating structure organization |
| 39 | GO:0043455 | 26303 | 10 | 1370 | 7 | 1.07E-07 | 3.54E-04 | regulation of secondary metabolic process |
| 40 | GO:0015629 | 26303 | 145 | 1370 | 25 | 1.25E-07 | 4.12E-04 | actin cytoskeleton |
| 41 | GO:0005976 | 26303 | 643 | 1370 | 66 | 1.25E-07 | 4.13E-04 | polysaccharide metabolic process |
| 42 | GO:0019747 | 26303 | 11 | 1370 | 7 | 2.81E-07 | 9.30E-04 | regulation of isoprenoid metabolic process |
| 43 | GO:0003779 | 26303 | 183 | 1370 | 28 | 3.01E-07 | 9.96E-04 | actin binding |
| 44 | GO:0030029 | 26303 | 206 | 1370 | 30 | 3.46E-07 | 0.0011455 | actin filament-based process |
| 45 | GO:0009699 | 26303 | 62 | 1370 | 15 | 4.80E-07 | 0.0015883 | phenylpropanoid biosynthetic process |
| 46 | GO:0030243 | 26303 | 177 | 1370 | 27 | 5.18E-07 | 0.0017136 | cellulose metabolic process |
| 47 | GO:0030036 | 26303 | 188 | 1370 | 28 | 5.29E-07 | 0.0017519 | actin cytoskeleton organization |
| 48 | GO:0006412 | 26303 | 1148 | 1370 | 99 | 5.65E-07 | 0.0018698 | translation |
| 49 | GO:0045834 | 26303 | 12 | 1370 | 7 | 6.44E-07 | 0.0021311 | positive regulation of lipid metabolic process |
| 50 | GO:0005875 | 26303 | 158 | 1370 | 25 | 6.73E-07 | 0.0022269 | microtubule associated complex |
| 51 | GO:0005975 | 26303 | 1917 | 1370 | 147 | 1.24E-06 | 0.0041082 | carbohydrate metabolic process |
| 52 | GO:0009808 | 26303 | 43 | 1370 | 12 | 1.29E-06 | 0.0042799 | lignin metabolic process |
| 53 | GO:0044085 | 26303 | 1329 | 1370 | 109 | 1.67E-06 | 0.0055143 | cellular component biogenesis |
| 54 | GO:0016757 | 26303 | 724 | 1370 | 68 | 2.03E-06 | 0.0067346 | transferase activity, transferring glycosyl groups |
| 55 | GO:0016759 | 26303 | 61 | 1370 | 14 | 2.27E-06 | 0.0075291 | cellulose synthase activity |
| 56 | GO:0016760 | 26303 | 61 | 1370 | 14 | 2.27E-06 | 0.0075291 | cellulose synthase (UDP-forming) activity |

| **Sequencing Orientation** | **Insert Size Mean (bp)** | **Insert Size STDDEV (bp)** | **Raw Read Length (bp)** | **Raw Read Pairs Number** | **Clean Read Pairs Number** | **Effective Depth*** |
| --- | --- | --- | --- | --- | --- | --- |
| Inward | 402 | 34 | 250 | 124,936,916 | 123,381,776 | 45.60 |

**Supplementary Table S23: The information on DNA PCR-free library**

* Effective Depth=Read length×2×Clean Read Pairs Number / Predicted genome size and Predicted genome size of 1,353 Mb is equal to the average of the two investigations of GCE and FindErros

For the preprocess, the low-quality reads (the percentage >40% and Q<13) were filtered using NGS QC Toolkit (version 2.3.3)[^1^](#_ENREF_1) with default parameters. Then, we have used FindErorrs, one of the scripts in ALLPATHS-LG (version r52488)[^2^](#_ENREF_2), to correct the filtered reads. Thus, we have predicted the genome size of 1,305,001,133 bp, identified repeat sequence of 54.5% (repeat time >1 and length >24 bp), detected SNP of 0.82% in genome and *k*mer coverage of 42X based on ALLPATHS-LG and the script of Kmer, one of the scripts in GCE (version 1.0.0 and ftp://ftp.genomics.org.cn/pub/gce/). Lastly, *De novo* assembling was preformed using DISCOVAR *de novo* (version r52488)[^3^](#_ENREF_3) with default parameters. The assembly results showed that contigs contained 1,971,146 sequences with more than 200 bp and N50 of contig is 4,828 bp. Total size of contig with more than 200 bp equal to 2,316,705,194 bp.

**Supplementary Table S24: The summary of genome survey for *D. jenkinsiana***

| Survey software | Genome size (Gb) | Kmer depth | Repeat rate | Heterozygosity rate |
| --- | --- | --- | --- | --- |
| Kmer script  (GCE) | 1.401 | 44 | 55.07% | 1.19% |
| FindErrors (ALLPATHS-LG) | 1.305 | 48 | 54.40% | 1.31% |

# Supplementary Table S25: Summary of single nucleotide polymorphisms

| SNP | CI | CD | HI | HD | Total |
| --- | --- | --- | --- | --- | --- |
| Transition | 130,584 | 132,548 | 96,683 | 122,877 | 482,692 |
| A-G | 63,272 | 64,181 | 46,965 | 59,464 | 233,882 |
| C-T | 67,312 | 68,367 | 49,718 | 63,413 | 248,810 |
| Transversion | 84,916 | 86,445 | 62,881 | 81,021 | 315,263 |
| A-C | 21,762 | 22,197 | 16,111 | 20,835 | 80,905 |
| A-T | 22,672 | 22,932 | 16,984 | 21,436 | 84,024 |
| C-G | 18,924 | 19,515 | 14,054 | 18,372 | 70,865 |
| G-T | 21,558 | 21,801 | 15,732 | 20,378 | 79,469 |
| Total | 215,500 | 218,993 | 159,564 | 203,898 | 797,955 |

# Supplementary Table S26: Summary of microsatellites in *Daemonorops jenkinsiana*

| Total sequences size (Mb) | SSR/Mb | Sequences | Mono- | Di- | Tri- | Tetra- | Penta- | Hexa- | Compound | Sequences with more than one SSR |
| --- | --- | --- | --- | --- | --- | --- | --- | --- | --- | --- |
| 83617731 | 5690.991 | 61569 | 12365 | 7135 | 5120 | 444 | 122 | 45 | 1827 | 5026 |

# Supplementary Table S27: The distribution of interrupted length

| **Interrupted Length (nt)** | **Number** | **%** | **Interrupted Length (nt)** | **Number** | **%** | **Interrupted Length (nt)** | **Number** | **%** | **Interrupted Length (nt)** | **Number** | **%** |
| --- | --- | --- | --- | --- | --- | --- | --- | --- | --- | --- | --- |
| 1 | 113 | 8.87% | 26 | 17 | 1.33% | 51 | 8 | 0.63% | 76 | 6 | 0.47% |
| 2 | 77 | 6.04% | 27 | 12 | 0.94% | 52 | 5 | 0.39% | 77 | 8 | 0.63% |
| 3 | 47 | 3.69% | 28 | 8 | 0.63% | 53 | 1 | 0.08% | 78 | 9 | 0.71% |
| 4 | 27 | 2.12% | 29 | 16 | 1.26% | 54 | 9 | 0.71% | 79 | 8 | 0.63% |
| 5 | 24 | 1.88% | 30 | 13 | 1.02% | 55 | 9 | 0.71% | 80 | 8 | 0.63% |
| 6 | 28 | 2.20% | 31 | 6 | 0.47% | 56 | 10 | 0.78% | 81 | 13 | 1.02% |
| 7 | 13 | 1.02% | 32 | 7 | 0.55% | 57 | 13 | 1.02% | 82 | 10 | 0.78% |
| 8 | 22 | 1.73% | 33 | 19 | 1.49% | 58 | 3 | 0.24% | 83 | 8 | 0.63% |
| 9 | 26 | 2.04% | 34 | 8 | 0.63% | 59 | 7 | 0.55% | 84 | 8 | 0.63% |
| 10 | 23 | 1.81% | 35 | 13 | 1.02% | 60 | 6 | 0.47% | 85 | 7 | 0.55% |
| 11 | 11 | 0.86% | 36 | 14 | 1.10% | 61 | 7 | 0.55% | 86 | 6 | 0.47% |
| 12 | 19 | 1.49% | 37 | 8 | 0.63% | 62 | 5 | 0.39% | 87 | 7 | 0.55% |
| 13 | 17 | 1.33% | 38 | 14 | 1.10% | 63 | 8 | 0.63% | 88 | 9 | 0.71% |
| 14 | 16 | 1.26% | 39 | 7 | 0.55% | 64 | 9 | 0.71% | 89 | 5 | 0.39% |
| 15 | 9 | 0.71% | 40 | 10 | 0.78% | 65 | 6 | 0.47% | 90 | 6 | 0.47% |
| 16 | 9 | 0.71% | 41 | 4 | 0.31% | 66 | 9 | 0.71% | 91 | 6 | 0.47% |
| 17 | 13 | 1.02% | 42 | 12 | 0.94% | 67 | 6 | 0.47% | 92 | 6 | 0.47% |
| 18 | 12 | 0.94% | 43 | 7 | 0.55% | 68 | 8 | 0.63% | 93 | 10 | 0.78% |
| 19 | 7 | 0.55% | 44 | 10 | 0.78% | 69 | 9 | 0.71% | 94 | 13 | 1.02% |
| 20 | 9 | 0.71% | 45 | 15 | 1.18% | 70 | 5 | 0.39% | 95 | 13 | 1.02% |
| 21 | 12 | 0.94% | 46 | 11 | 0.86% | 71 | 8 | 0.63% | 96 | 12 | 0.94% |
| 22 | 8 | 0.63% | 47 | 9 | 0.71% | 72 | 12 | 0.94% | 97 | 5 | 0.39% |
| 23 | 17 | 1.33% | 48 | 7 | 0.55% | 73 | 1 | 0.08% | 98 | 7 | 0.55% |
| 24 | 14 | 1.10% | 49 | 12 | 0.94% | 74 | 4 | 0.31% | 99 | 6 | 0.47% |
| 25 | 15 | 1.18% | 50 | 8 | 0.63% | 75 | 9 | 0.71% | 100+ | 46 | 3.61% |

# Supplementary Table S28: Frequency of di- to hexa-nucleotide repeat motifs

| **Repeat motif** | | **Number** | **%** | **Repeat motif** | | **Number** | **%** |
| --- | --- | --- | --- | --- | --- | --- | --- |
| Di-nucleotide | AC/GT | 964 | 13.51% | Penta-nucleotide | AAAGT/ACTTT | 1 | 0.82% |
|  | AG/CT | 4668 | 65.42% |  | AAATT/AATTT | 1 | 0.82% |
|  | AT/AT | 1404 | 19.68% |  | AACAT/ATGTT | 1 | 0.82% |
|  | CG/CG | 99 | 1.39% |  | AACCC/GGGTT | 2 | 1.64% |
| Tri-nucleotide | AAC/GTT | 337 | 6.58% |  | AACTC/AGTTG | 42 | 34.43% |
|  | AAG/CTT | 822 | 16.05% |  | AAGAG/CTCTT | 12 | 9.84% |
|  | AAT/ATT | 528 | 10.31% |  | AAGGG/CCCTT | 3 | 2.46% |
|  | ACC/GGT | 321 | 6.27% |  | AATCC/ATTGG | 2 | 1.64% |
|  | ACG/CGT | 234 | 4.57% |  | ACCTC/AGGTG | 1 | 0.82% |
|  | ACT/AGT | 46 | 0.90% |  | ACGAG/CGTCT | 1 | 0.82% |
|  | AGC/CTG | 503 | 9.82% |  | ACGGC/CCGTG | 1 | 0.82% |
|  | AGG/CCT | 1173 | 22.91% |  | ACGGG/CCCGT | 1 | 0.82% |
|  | ATC/ATG | 286 | 5.59% |  | ACTCC/AGTGG | 1 | 0.82% |
|  | CCG/CGG | 870 | 16.99% |  | AGAGG/CCTCT | 9 | 7.38% |
| Tetra-nucleotide | AAAC/GTTT | 22 | 4.95% |  | AGATG/ATCTC | 1 | 0.82% |
|  | AAAG/CTTT | 101 | 22.75% |  | AGGGG/CCCCT | 1 | 0.82% |
|  | AAAT/ATTT | 109 | 24.55% |  | ATCCC/ATGGG | 1 | 0.82% |
|  | AACC/GGTT | 5 | 1.13% |  | CCCCG/CGGGG | 1 | 0.82% |
|  | AACG/CGTT | 2 | 0.45% | Hexa-nucleotide | AAAAAC/GTTTTT | 1 | 2.22% |
|  | AAGC/CTTG | 3 | 0.68% |  | AAAAAG/CTTTTT | 6 | 13.33% |
|  | AAGG/CCTT | 10 | 2.25% |  | AAAAAT/ATTTTT | 3 | 6.67% |
|  | AAGT/ACTT | 1 | 0.23% |  | AAAGAC/CTTTGT | 2 | 4.44% |
|  | AATC/ATTG | 4 | 0.90% |  | AAAGGC/CCTTTG | 2 | 4.44% |
|  | AATG/ATTC | 4 | 0.90% |  | AACAAT/ATTGTT | 1 | 2.22% |
|  | AATT/AATT | 2 | 0.45% |  | AACACC/GGTGTT | 3 | 6.67% |
|  | ACAG/CTGT | 1 | 0.23% |  | AACCCT/AGGGTT | 1 | 2.22% |
|  | ACAT/ATGT | 58 | 13.06% |  | AAGAGG/CCTCTT | 2 | 4.44% |
|  | ACCG/CGGT | 6 | 1.35% |  | AAGGAG/CCTTCT | 5 | 11.11% |
|  | ACCT/AGGT | 4 | 0.90% |  | AAGGGC/CCCTTG | 1 | 2.22% |
|  | ACGC/CGTG | 5 | 1.13% |  | AATGGC/ATTGCC | 1 | 2.22% |
|  | ACGG/CCGT | 11 | 2.48% |  | ACACCC/GGGTGT | 1 | 2.22% |
|  | ACGT/ACGT | 2 | 0.45% |  | ACATAT/ATATGT | 3 | 6.67% |
|  | ACTC/AGTG | 2 | 0.45% |  | ACCATC/ATGGTG | 1 | 2.22% |
|  | AGAT/ATCT | 21 | 4.73% |  | ACCTCC/AGGTGG | 1 | 2.22% |
|  | AGCC/CTGG | 2 | 0.45% |  | ACCTCG/AGGTCG | 1 | 2.22% |
|  | AGCG/CGCT | 28 | 6.31% |  | ACGAGG/CCTCGT | 1 | 2.22% |
|  | AGGC/CCTG | 4 | 0.90% |  | ACTCCG/AGTCGG | 1 | 2.22% |
|  | AGGG/CCCT | 16 | 3.60% |  | ACTGCT/AGCAGT | 1 | 2.22% |
|  | ATCC/ATGG | 9 | 2.03% |  | AGAGCT/AGCTCT | 1 | 2.22% |
|  | ATCG/ATCG | 3 | 0.68% |  | AGATCT/AGATCT | 1 | 2.22% |
|  | ATGC/ATGC | 7 | 1.58% |  | AGATGG/ATCTCC | 1 | 2.22% |
|  | CCCG/CGGG | 2 | 0.45% |  | AGCCTG/AGGCTC | 1 | 2.22% |
| Penta-nucleotide | AAAAC/GTTTT | 5 | 4.10% |  | AGGATC/ATCCTG | 1 | 2.22% |
|  | AAAAG/CTTTT | 15 | 12.30% |  | AGGATG/ATCCTC | 1 | 2.22% |
|  | AAAAT/ATTTT | 19 | 15.57% |  | AGGGCG/CCCTCG | 1 | 2.22% |
|  | AAAGG/CCTTT | 1 | 0.82% |  |  |  |  |

# Supplementary Table S29: The SSR distribution in different regions of unigenes

| Type | | Amount | % |
| --- | --- | --- | --- |
| Independent region | CDS | 4,439 | 29.74% |
|  | 5′UTR | 4,515 | 30.25% |
|  | 3′UTR | 4,937 | 33.07% |
|  | UTR* | 860 | 5.76% |
| Two adjacent regions | CDS and 5′UTR | 94 | 0.63% |
|  | CDS and 3′UTR | 68 | 0.46% |
|  | CDS and UTR | 15 | 0.10% |

*: UTR represents the untranslated region.

# Supplementary Table S30: The whole parameters of Primer3

| Primer3 File |  |
| --- | --- |
| P3_FILE_TYPE | settings |
| P3_FILE_ID | P3 Settings |
| PRIMER_FIRST_BASE_INDEX | 1 |
| PRIMER_TASK | generic |
| PRIMER_NUM_RETURN | 5 |
| PRIMER_PICK_LEFT_PRIMER | 1 |
| PRIMER_PICK_INTERNAL_OLIGO | 0 |
| PRIMER_PICK_RIGHT_PRIMER | 1 |
| PRIMER_PICK_ANYWAY | 1 |
| PRIMER_THERMODYNAMIC_PARAMETERS_PATH | /home/bioinfor/primer3_config/ |
| PRIMER_TM_FORMULA | 1 |
| PRIMER_MIN_TM | 55 |
| PRIMER_OPT_TM | 60 |
| PRIMER_MAX_TM | 65 |
| PRIMER_PAIR_MAX_DIFF_TM | 5 |
| PRIMER_WT_TM_LT | 0 |
| PRIMER_WT_TM_GT | 0 |
| PRIMER_PAIR_WT_DIFF_TM | 0 |
| PRIMER_MIN_SIZE | 18 |
| PRIMER_OPT_SIZE | 20 |
| PRIMER_MAX_SIZE | 22 |
| PRIMER_WT_SIZE_LT | 0 |
| PRIMER_WT_SIZE_GT | 0 |
| PRIMER_MIN_GC | 30 |
| PRIMER_MAX_GC | 70 |
| PRIMER_WT_GC_PERCENT_LT | 0 |
| PRIMER_WT_GC_PERCENT_GT | 0 |
| PRIMER_THERMODYNAMIC_OLIGO_ALIGNMENT | 1 |
| PRIMER_MAX_SELF_ANY | 8 |
| PRIMER_WT_SELF_ANY | 0 |
| PRIMER_MAX_SELF_ANY_TH | 45 |
| PRIMER_WT_SELF_ANY_TH | 123.2 |
| PRIMER_MAX_SELF_END | 3 |
| PRIMER_WT_SELF_END | 0 |
| PRIMER_MAX_SELF_END_TH | 35 |
| PRIMER_WT_SELF_END_TH | 302.4 |
| PRIMER_PAIR_MAX_COMPL_ANY | 8 |
| PRIMER_PAIR_WT_COMPL_ANY | 0 |
| PRIMER_PAIR_MAX_COMPL_ANY_TH | 45 |
| PRIMER_PAIR_WT_COMPL_ANY_TH | 123.2 |
| PRIMER_PAIR_MAX_COMPL_END | 3 |
| PRIMER_PAIR_WT_COMPL_END | 0 |
| PRIMER_PAIR_MAX_COMPL_END_TH | 35 |
| PRIMER_PAIR_WT_COMPL_END_TH | 302.4 |
| PRIMER_MAX_HAIRPIN_TH | 24 |
| PRIMER_WT_HAIRPIN_TH | 672 |
| PRIMER_MAX_END_STABILITY | 9 |
| PRIMER_WT_END_STABILITY | 1 |
| PRIMER_LOWERCASE_MASKING | 0 |
| PRIMER_MAX_POLY_X | 4 |
| PRIMER_MAX_NS_ACCEPTED | 0 |
| PRIMER_WT_NUM_NS | 0 |
| PRIMER_MAX_END_GC | 5 |
| PRIMER_GC_CLAMP | 0 |
| PRIMER_LIBERAL_BASE | 1 |
| PRIMER_LIB_AMBIGUITY_CODES_CONSENSUS | 0 |
| PRIMER_MIN_QUALITY | 0 |
| PRIMER_MIN_END_QUALITY | 0 |
| PRIMER_QUALITY_RANGE_MIN | 0 |
| PRIMER_QUALITY_RANGE_MAX | 100 |
| PRIMER_WT_SEQ_QUAL | 0 |
| PRIMER_WT_END_QUAL | 0 |
| PRIMER_SEQUENCING_LEAD | 50 |
| PRIMER_SEQUENCING_SPACING | 500 |
| PRIMER_SEQUENCING_INTERVAL | 250 |
| PRIMER_SEQUENCING_ACCURACY | 20 |
| PRIMER_OUTSIDE_PENALTY | 0 |
| PRIMER_INSIDE_PENALTY | -1 |
| PRIMER_WT_POS_PENALTY | 0 |
| PRIMER_SALT_MONOVALENT | 50 |
| PRIMER_SALT_CORRECTIONS | 1 |
| PRIMER_SALT_DIVALENT | 1.5 |
| PRIMER_DNTP_CONC | 0.6 |
| PRIMER_DNA_CONC | 50 |
| PRIMER_PRODUCT_MIN_TM | -1000000 |
| PRIMER_PRODUCT_OPT_TM | 0 |
| PRIMER_PRODUCT_MAX_TM | 1000000 |
| PRIMER_PAIR_WT_PRODUCT_TM_LT | 0 |
| PRIMER_PAIR_WT_PRODUCT_TM_GT | 0 |
| PRIMER_PRODUCT_OPT_SIZE | 0 |
| PRIMER_PAIR_WT_PRODUCT_SIZE_LT | 0 |
| PRIMER_PAIR_WT_PRODUCT_SIZE_GT | 0 |
| PRIMER_PAIR_WT_PR_PENALTY | 1 |
| PRIMER_PAIR_WT_IO_PENALTY | 0 |
| PRIMER_INTERNAL_MIN_TM | 57 |
| PRIMER_INTERNAL_OPT_TM | 60 |
| PRIMER_INTERNAL_MAX_TM | 63 |
| PRIMER_INTERNAL_WT_TM_LT | 1 |
| PRIMER_INTERNAL_WT_TM_GT | 1 |
| PRIMER_INTERNAL_MIN_SIZE | 18 |
| PRIMER_INTERNAL_OPT_SIZE | 20 |
| PRIMER_INTERNAL_MAX_SIZE | 27 |
| PRIMER_INTERNAL_WT_SIZE_LT | 1 |
| PRIMER_INTERNAL_WT_SIZE_GT | 1 |
| PRIMER_INTERNAL_MIN_GC | 20 |
| PRIMER_INTERNAL_MAX_GC | 80 |
| PRIMER_INTERNAL_OPT_GC_PERCENT | 50 |
| PRIMER_INTERNAL_WT_GC_PERCENT_LT | 0 |
| PRIMER_INTERNAL_WT_GC_PERCENT_GT | 0 |
| PRIMER_INTERNAL_MAX_SELF_ANY | 12 |
| PRIMER_INTERNAL_WT_SELF_ANY | 0 |
| PRIMER_INTERNAL_MAX_SELF_END | 12 |
| PRIMER_INTERNAL_WT_SELF_END | 0 |
| PRIMER_INTERNAL_MAX_POLY_X | 5 |
| PRIMER_INTERNAL_MAX_NS_ACCEPTED | 0 |
| PRIMER_INTERNAL_MIN_QUALITY | 0 |
| PRIMER_INTERNAL_WT_END_QUAL | 0 |
| PRIMER_INTERNAL_WT_SEQ_QUAL | 0 |
| PRIMER_INTERNAL_SALT_MONOVALENT | 50 |
| PRIMER_INTERNAL_SALT_DIVALENT | 1.5 |
| PRIMER_INTERNAL_DNA_CONC | 50 |
| PRIMER_INTERNAL_DNTP_CONC | 0 |

# Supplementary Table S31: List of 168 primer pairs used in the study for amplification

(Please see the supplementary Excel file)

# Supplementary Table S32: The list of 25 accessions used in this study

| **Code** | **Accession name** | **Scientific name** |
| --- | --- | --- |
| 1 | Chang bing huang teng | *Daemonorops longistipes* |
| 2 | Xia ye huang teng | *D. angustifolia* |
| 3 | Huang teng | *D. jenkinsiana* |
| 4 | Huang teng species 11(Unnamed) | *D. sp.11* |
| 5 | Meng bang sheng teng | *Calamus viminalis* |
| 6 | Ban na sheng teng | *C. nambariensis var. xishuangbannaensis* |
| 7 | He lin sheng teng | *C. balansaeanus var. castaneolepis* |
| 8 | Meng bang sheng teng bian zhong | *C. viminalis var. faseiculatus* |
| 9 | Duo guo sheng teng | *C. walkeri* |
| 10 | Ying jiang sheng teng | *C. nambariensis var. yingjiangensis* |
| 11 | Dian nan sheng teng | *C. ﻿henryanus* |
| 12 | Xi jia sheng teng | *C. saeius* |
| 13 | Xi jing sheng teng | *C. exilis* |
| 14 | Gao di sheng teng | *C. nambariensis var. alpinus* |
| 15 | Tuo yuan guo sheng teng | *C. guruba* |
| 16 | Meng la sheng teng | *C. flagellum var. karinensis* |
| 17 | Ban ling sheng teng | *C. banlingensis* |
| 18 | Dian mian sheng teng | *C. erectus var. birmanicus* |
| 19 | Xi sheng teng | *C. filiformis* |
| 20 | Sheng teng species 5 (Unnamed) | *C. sp.5* |
| 21 | Dan ye sheng teng | *C. simplicifolius* |
| 22 | Sheng teng species 1 (Unnamed) | *C. sp.1* |
| 23 | Sheng teng species 2 (Unnamed) | *C. sp.2* |
| 24 | Sheng teng species 3 (Unnamed) | *C. sp.3* |
| 25 | Sheng teng species 4 (Unnamed) | *C. sp.4* |

# Supplementary Table S33: The genetic distance between the 25 accessions used in this study

# Supplementary Table S34. The expression value of candidate reference genes in 8 samples

|  | CI_rep1 | CI_rep2 | CD_rep1 | CD_rep2 | HI_rep1 | HI_rep2 | HD_rep1 | HD_rep2 | Mean | SD | CV | Annotation |
| --- | --- | --- | --- | --- | --- | --- | --- | --- | --- | --- | --- | --- |
| TR97697_c2_g9 | 508.53 | 482 | 577 | 461.7 | 585 | 533 | 399.99 | 355 | 487.7775 | 81.01017 | 0.16608 | γ-tubulin |
| TR53058_c0_g1 | 260 | 210 | 390 | 374 | 295 | 313 | 401 | 313 | 319.5 | 66.33034 | 0.207607 | Actin |
| TR90587_c1_g5 | 13867 | 12447 | 13503 | 12853.01 | 12746.01 | 12002 | 11224.01 | 9140 | 12222.75 | 1493.485 | 0.122189 | Ubiquitin-conjugating enzyme |
